# How cognitive biases affect winning probability perception in beach volleyball experts

**DOI:** 10.1038/s41598-025-17770-z

**Published:** 2025-09-11

**Authors:** Sandra Ittlinger, Steffen Lang, Antonia Schubert, Markus Raab

**Affiliations:** 1 Department of Performance Psychology, German Sport University Cologne, Cologne, Germany; 2 TUM School of Medicine and Health, Chair of Performance Analysis and Sports Informatics, Technical University of Munich, Munich, Germany; 3 Institute of Sports Science, Department of Sport Psychology and Research Methods, Eberhard Karls University Tübingen, Tübingen, Germany

**Keywords:** Decision making in Sport, Winning Probability Perception, Optimism bias, Confirmation bias, Beach Volleyball, Elite sport, Human behaviour, Psychology

## Abstract

In professional beach volleyball, the belief in “never give up” is deeply ingrained, but its strategic implications remain underexplored. This study employs a mixed-methods approach, combining sport psychology and sport informatics, to assess the perception of set-winning probabilities (SWPs) in beach volleyball, as this is a crucial factor for strategic in-game decisions and improved performance. We examined cognitive biases and adaptive strategies influencing SWP estimations in scenarios of substantial trailing or leading. Forty-three members of the German beach volleyball national team estimated SWPs for 60 scores, responded to questions on game tactics, and completed questionnaires measuring optimism, pessimism, confirmation bias, and the sunk cost fallacy. Empirical SWPs were calculated from a dataset of 6,571 matches. Results revealed that participants significantly overestimated SWPs when trailing and underestimated them when leading. Optimism and confirmation bias significantly shaped these estimations. Notably, confirmation bias had a dual role: in trailing scenarios, it amplified overestimation, causing players to underestimate their disadvantage, while in leading scenarios, it improved accuracy by focusing on the likelihood of victory. Players were more likely to recall situations reinforcing the belief that “We (can still) win”. These findings highlight the psychological and strategic complexities of SWP estimations in competitive beach volleyball.

**Supplementary Information:**

The online version contains supplementary material available at 10.1038/s41598-025-17770-z.

## Introduction

In professional sports, questions that appear simple at first glance, such as “Can we still win this game?“, often require interdisciplinary approaches to uncover meaningful answers. The integration of sport psychology and sport informatics^1^ provides a framework for examining such phenomena by combining psychological theories, empirical data, and idiosyncratic insights. Our study focuses on solving a practical problem emerging from beach volleyball: understanding how cognitive biases influence the perception of the set-winning probability (SWP) at a certain moment in-game and their implications for strategic decision-making during competition. This mixed-methods approach highlights the potential of interdisciplinary work to bridge the gap between theoretical concepts and practical needs in professional sports, enhancing athletes’ performance.

The phrase “never give up” is frequently seen as an example of unwavering optimism, sportsmanship, and dedication in the world of elite sports. Players are celebrated for their relentless perseverance, fighting for every point, and pushing through even the most difficult situations. However, do moments exist when a strategic retreat or a calculated adjustment might serve them better? The beach volleyball performance analysis team of the German Volleyball Federation posed this question, anticipating that insights gained from data-based knowledge could significantly impact the teams’ success in competition. Our study seeks to clarify the complex interplay between the moral valuation of perseverance and the practical realities of statistical data. By examining the behavior and decision-making processes of elite players and coaches, we aim to determine whether the heroic effort to “never give up” leads to a problematic discrepancy between the perception of winning probabilities and their empirical equivalents.

When making decisions, people do not always follow the guidelines provided by formal cost-benefit models, probability reasoning, logic, or prudent considerations but instead rely on more intuitive approaches^2–5^. Thereby, people exhibit the same typical tendencies in the way they take in and process information in order to judge and make decisions under a wide range of different conditions. These tendencies are very specific and systematic rather than random^4,6^ and are referred to as cognitive biases^5^. In general, cognitive biases are defined as widespread tendencies that systematically distort information processing, often leading to inaccurate or suboptimal outcomes^7^.

In the field of sports, decision-making is frequently shaped by such biases. For example, in ice hockey, the tactic of pulling the goalie when trailing late in the game, which means removing the goalkeeper in favor of an extra attacker to increase the chances of scoring, is a tactical approach often executed too late by coaches^8^. This can be interpreted as loss aversion, where the fear of conceding an empty-net goal outweighs the potential benefits of increased offensive pressure, reflecting a bias towards avoiding immediate losses rather than optimizing for long-term success^9^. Another study on base rates and sequential decisions in beach volleyball^10^ demonstrated that players’ and coaches’ perceptions are biased when rating performances. Here, base rate estimates differ when looking at successful sequences because multiple hits in a row produce so-called hot hand beliefs. Other reviews and meta-analyses further suggest that perception in sports often diverges significantly from objective reality^11–13^.

In a recent study by Lillich and colleagues^14^, the authors found that around 60% of beach volleyball games between equally skilled teams ended after two sets rather than three. They interpreted this result as the winners of the first set expended more effort in the second set than the losers. Whether this effect appears due to effort, strategy changes, different team conditions, simple luck, or variance in competitive behavior requires further investigation^15–17^. The recommendation for a team losing the first set cannot be to give up, but to explore the causes, provide insights like winning probabilities, and support strategic adjustments. For instance, beach volleyball teams frequently increase serving risk when trailing significantly, aiming to reduce the opponents’ side-out chances^18^. However, the effectiveness and timing of such a strategy remain unknown, which has led to the formation of subjective and possibly biased beliefs that require further examination.

A possible explanation for the “never give up” mentality in players could be the optimism bias, which describes a tendency to evaluate future events positively and underestimate the probability of negative outcomes^19,20^. Optimism as a trait was found to correlate positively with success in sports^21^, sport confidence^22^, superior physical and psychological health^23^, as well as coping strategies^24^. Specifically, behavioral disengagement, characterized by the discontinuation of efforts to address a stressor, has been negatively correlated with optimism^24^, indicating that individuals with higher levels of optimism are less likely to abandon their goals. Athletes, in particular, often exhibit higher levels of optimism compared to the general population^25^, possibly due to the demands of competitive sports that require resilience and a positive outlook^26^. However, optimism is not entirely beneficial, as it can, under certain circumstances, lead to negative outcomes^23^. In the context of beach volleyball, where no specific studies have yet been conducted, optimism may manifest as a belief in one’s ability to win despite adverse statistical probabilities. We assume that players who score high in optimism as trait-like individual differences are less inclined to give up on their goals. Importantly, while optimism can be considered a stable trait-like individual difference^27^, it has been found to fluctuate based on levels of self-esteem, confidence, social resources, and controllable versus uncontrollable outcomes^28–30^. These situational factors may also play a crucial role when an athlete assesses their chances of success. The perceived probability of victory may vary based on whether the athlete is in the lead or trailing during a match. In such scenarios, an athlete with high state optimism might maintain a belief in their ability to come back from a disadvantage, whereas someone with lower state optimism might be more inclined to disengage.

Although optimism bias has been thoroughly investigated in sports, establishing a solid foundation for analyzing its correlation with persistence in athletic objectives^19,20^, various other cognitive biases may affect players’ decision-making. These biases, including confirmation bias, the tendency to prioritize information that validates existing beliefs^31,32^, and the sunk cost fallacy, the tendency to persist in a course of action due to previous investments^33,34^, have garnered relatively less focus in this field. Despite the limited empirical research on these additional biases in sports, we incorporate them into our analysis to investigate their potential effects in an exploratory manner, rather than developing specific hypotheses.

In the current study, we apply a mixed-methods approach that aims to examine psychological factors that influence athletic performance in estimating the SWP as a crucial factor of in-game tactical adjustments. To achieve this, we integrate SWPs at specific scores in beach volleyball, calculated from a large empirical dataset, with players’ probability estimations, which may be affected by optimism or other biases. This is supported by a survey of idiosyncratic insights into players’ thinking in the case of substantial trailing and leading. So far, there is no reliable information on how coaches and players perceive SWPs. We seek to describe for the first time how accurately experts estimate the SWP at specific scores in beach volleyball competitions. In principle, we expect two different effects of the optimism bias on perceiving winning probabilities. First, a general trait-like bias that is independent of the specific situation, players with high optimism bias overestimate winning probabilities and thus a tactical change most often occurs too late. Second, and a reasonable alternative assumption is, if the findings of existing studies on fluctuating optimism apply to our study’s sample and task, scenarios such as trailing in a beach volleyball match may activate state-like optimism. This could mean that optimism effects on SWP estimations are present or particularly strong only when trailing. To achieve this, we specified three questions:

1. How do experts estimate the probability of winning the set at certain scores in beach volleyball competition?
2. Which strategies do experts pursue when trailing or leading?
3. Do cognitive biases influence the estimations and encourage persistence in challenging situations in beach volleyball competition?

## Methods

### Participants

Thirty-three members of the German beach volleyball national team squad and ten German beach volleyball national team coaches participated in this study and provided written informed consent (Table 1). In addition, players aged 16 and 17 were required to provide informed consent of a guardian. Data was collected between June 15 and October 15, 2024 using the online platform Unipark (www.unipark.de). On average, players spent 17 min completing the online survey. Because only the German national-level squads were accessible, a priori power calculations for sample size were not feasible^35^; instead, we treated this cohort as a convenience sample of elite athletes and coaches, whose responses we believe reasonably represent patterns in top‑level beach volleyball internationally. Each player was contacted individually by our study manager, and the survey was conducted in German. Ethics approval was obtained prior to data collection by the local university’s ethics committee (082/2024), and the study procedure was in line with the APA 7th Edition standards for ethical engagement with participants.

**Table 1:** Characteristics of the participants. Shown are the mean and standard deviation within the group alongside minimum and maximum values.

	Players									Coaches		
Sex	M (*n* = 19)			F (*n* = 14)			All (*n* = 33)			All (*n* = 10)		
	Mean ± SD	min	max	Mean ± SD	min	max	Mean ± SD	min	max	Mean ± SD	min	max
Age [years]	20.8 ± 2.9	16	26	22.3 ± 4.0	17	32	21.5 ± 3.5	16	32	42.5 ± 7.9	30	58
Experience [years]	4.2 ± 2.4	1	10	5.4 ± 4.1	1	15	4.7 ± 3.2	1	15	21.6 ± 9.5	10	38
Training [h]	13.6 ± 4.9	7	24	12.1 ± 3.1	6	18	13.0 ± 4.2	6	24	20.1 ± 3.3	14	25

Coaches are not split by sex, as only one female coach was part of the study. Experience refers to years as an elite player and/or coach.

### Materials and procedure

In this study, we focused on examining coaches’ and players’ estimates of winning probabilities at specific scores and whether cognitive biases can partly explain those estimates. To answer question 1 on how experts estimate the probability of winning the set at specific scores, we presented players and coaches with a questionnaire featuring 60 different scores from a beach volleyball match, asking them to estimate the likelihood of winning the set. Participants were asked to imagine serving in either the first (36 times) or third (24 times) set against an opponent of equal skill. They assessed their perceived winning probabilities on a scale from 0 to 100%. For selecting the scores to ask, we conducted an extensive examination of a dataset provided by the match-analytics team of the German volleyball federation. A cooperation contract with the federation allowed us to use the archive data from the national scouting department. This dataset consists of 6571 matches performed between 2017 and 2024 on the highest level in beach volleyball (4- and 5-Star tournaments before 2022; since 2022 Beach Pro Tour Challenger, Elite 16, Finals; Olympic Games; and World Championships). The number of matches is nearly equal between males and females, with 3287 and 3284 matches respectively. Therefore, we plotted the scores’ distribution appearing in these matches from the serving team’s point of view. From this, we excluded all scores with a lower probability of occurrence than 7.5% to ensure that we focus on the most occurring game situations. Half of the selected scores (30) were randomly chosen from situations where a side switch occurs (every 7 points in set 1 and every 5 points in set 3), whereas the scores at the technical timeout, occurring after 21 rallies in set one and two, were included completely, and the other half of the scores (30) were randomly selected from all remaining scores where no side switch occurs. This procedure was performed to ensure a good distribution of scores between those with side switch, where a team has enough time to talk to each other and adjust their actual tactical approach, and without. Supplementary Figure S1 visualizes the selected scores alongside the excluded scores and the distribution.

To address question 2 on which strategies players and coaches pursue when trailing or leading, the participants were asked four questions about their game tactics for different scores through a qualitative survey following the SWP estimates assessment. The four questions were (1) Are specific strategies used to maintain a large lead (> 3 points)? And if yes, which? (2) What does a large lead or a high winning probability mean to you or your team? (3) Are specific strategies used to overcome a deficit? And if yes, which? (4) What does a large deficit or a low winning probability mean to you or your team?

Subsequently, for question 3, whether cognitive biases influence probability estimations and encourage persistence in challenging situations, participants completed questionnaires assessing optimism, pessimism, confirmation bias, and the sunk cost fallacy.

The *optimism and pessimism bias* was measured through the Life Orientation Test-Revised (LOT-R) which consists of ten items: three items assessing optimism, three assessing pessimism, and four filler items^36^. An optimism and a pessimism subscale of the LOT has been separated and found to correlate differently with criterion variables^37,38^. We decided to use both scales of optimism and pessimism as well as the total score of LOT-R as the trailing and leading situation manipulated may allow us to differentiate effects on sub-scale or total score level. Each item like “In uncertain times, I usually expect the best” is rated on a 5-point Likert scale ranging from 0 (strongly disagree) to 4 (strongly agree). The scale demonstrated good internal consistency with Cronbach’s alpha of α = 0.70 for the optimism subscale.

The *confirmation bias* was assessed through the confirmation inventory which consists of ten items in total^31^. Each item, such as “I only need a little information to reach a good decision” was rated on a 5-point Likert scale ranging from 1 (strongly disagree) to 5 (strongly agree). The Cronbach’s alpha coefficient of this scale shows acceptable reliability with α = 0.65^39^.

The *sunk cost fallacy* was measured through the sunk cost effect scale (SCE-8)^40^ which presents participants with eight hypothetical scenarios, such as “You have an investment strategy that you have developed over several months. It is not working and you are losing money, but there is no way for you to recover the lost effort put into developing the strategy”. For each item, there is a 6-point Likert scale for which the two alternatives (in this item’s case “start afresh” and “keep going”) are written over the leftmost and rightmost points^34,41^. This scale demonstrated high internal consistency, with Cronbach’s alpha of α = 0.75.

### Data processing

We performed data processing in two steps. The first step involved excluding faulty data, while the second step addressed outliers using a customized approach tailored to our survey. This customization was necessary because no standardized method adequately fits our data, given that some values could be statistically labeled outliers but are based on valid reasoning of the individual participant.

In step one, we excluded the data of three participants who either provided unreliable answers or failed to complete the survey. Additionally, errors in data acquisition caused by the online survey platform Unipark (www.unipark.de) affected four participants, resulting in the exclusion of 13 score estimates. In step two, we implemented a customized approach to handle outliers. This approach was designed to retain participant estimates that were close to the empirical SWP, even if they significantly differed from the rest of the sample. Standardized approaches, such as winsorizing or trimming, would penalize these reasonable estimates and potentially distort our findings. To address this, we excluded outliers for each estimated score based on two criteria: first, estimates that differed by more than two standard deviations from the sample mean. Second, estimates that deviated by more than 25% above or below the empirical SWP were excluded. This approach ensured that estimates near the empirical SWP were preserved, even when they differed considerably from the sample’s overall pattern.

Additionally, we present complete results for two alternative outlier handling approaches used in step two in the Supplementary Material: a standardized winsorizing approach and one without any outlier handling. A comparison of the three approaches can be found in the Supplementary Material.

### Data analysis

To analyze question 1, participants were divided into three groups: male players, female players, and coaches. Coaches were not split by gender, as only one female coach participated in the survey. We visualized the estimated probabilities for each participant group and assessed scores using box plots, which were compared to the empirically measured probabilities from our dataset. Scores were categorized into five groups based on the point difference (D): a tie (D_0_), a moderate leading (D_+2_), and a moderate trailing (D_-2_) with a score difference ± 1 or 2, a substantial leading (D_+3_), and a trailing (D_-3_) (± 3 or more). For these categories, we tested whether participant groups differed in their estimation precision. Normality was assessed using the Shapiro-Wilk test. For normally distributed data, Welch’s t-test was applied due to its robustness to unequal variances. For non-normally distributed data, Mann-Whitney U tests were used. Effect sizes were calculated using Cohen’s *d* for both t-tests and Mann-Whitney U tests, with the latter derived from the z-value transformation for effect size calculations from non-parametric tests^42^. To control Type I error due to multiple comparisons, we applied the Bonferroni correction, adjusting the significance threshold to α / *m*, where *m* represents the number of comparisons and α the significance level. We used *α* = 0.05, 0.01, and 0.001 as different significance thresholds. To calculate the empirical SWP, we utilized the collected match data including the serving team, current score, whether the serving team won the set, player gender, and set number. For each score, we computed the frequency of occurrences and the percentage of cases in which the serving team won the set under the given conditions.

To identify the patterns of meaning across the data for question 2, the data were analyzed using a thematic analysis navigating the process of coding, discussion, and thematic development^43^. Braun and Clarke’s^44^ six recursive phases of familiarization, coding, generating initial themes, reviewing and developing themes, refining, defining and naming themes, and writing up were followed. Guiding questions included, e.g., “*What strategies are used to maintain a lead of 3 or more points?”*, “*What does a high lead or high win probability mean for you or your team?”*, “*What strategies are used to compensate for a deficit of 3 or more points?”*, “*What does a high deficit or a low probability of winning mean for you or your team?”.*

To address question 3, we analyzed the distribution of the tested biases across the three participant groups and tested for group differences. To evaluate the impact of these biases on SWP estimation discrepancy (SWP-ED), we conducted single linear regression analyses for each score category, calculating Pearson correlation coefficients (*r*) to quantify the strength and direction of the relationships. Additionally, we employed robust linear modeling (RLM)^45^ using Huber’s T estimator^46^ to examine the combined effects of all assessed biases on SWP-ED across the five score categories. RLM was chosen due to its advantages over ordinary least squares regression in the presence of potential outliers, offering more stable coefficient estimates. In line with this, we applied only the first data-cleaning step (removal of implausible responses) without any further outlier correction, as RLM inherently accounts for outliers. As RLM does not yield a standard coefficient of determination (*R*^2^), we report the squared Pearson correlation between observed and predicted values (*r*^2^) as a descriptive pseudo-*R*^2^. For transparency, both analyses, single linear regression and RLM, were also applied to the full sample presented in the Supplementary Material.

This study was conceptually grounded within a pragmatist framework, which perceives “truth” not as an absolute but as a functional outcome that aids in problem solving, and improves actions and interactions^47^. In this view, knowledge is seen as a collective creation, emerging from the interactions between participants as well as between researchers and participants. This approach led us to embrace a subjectivist and transactional epistemology, acknowledging the dynamic and co-creative nature of knowledge construction^48,49^.

## Results

### How do experts estimate the probability of winning the set at certain scores in beach volleyball competition?

**Fig. 1:**
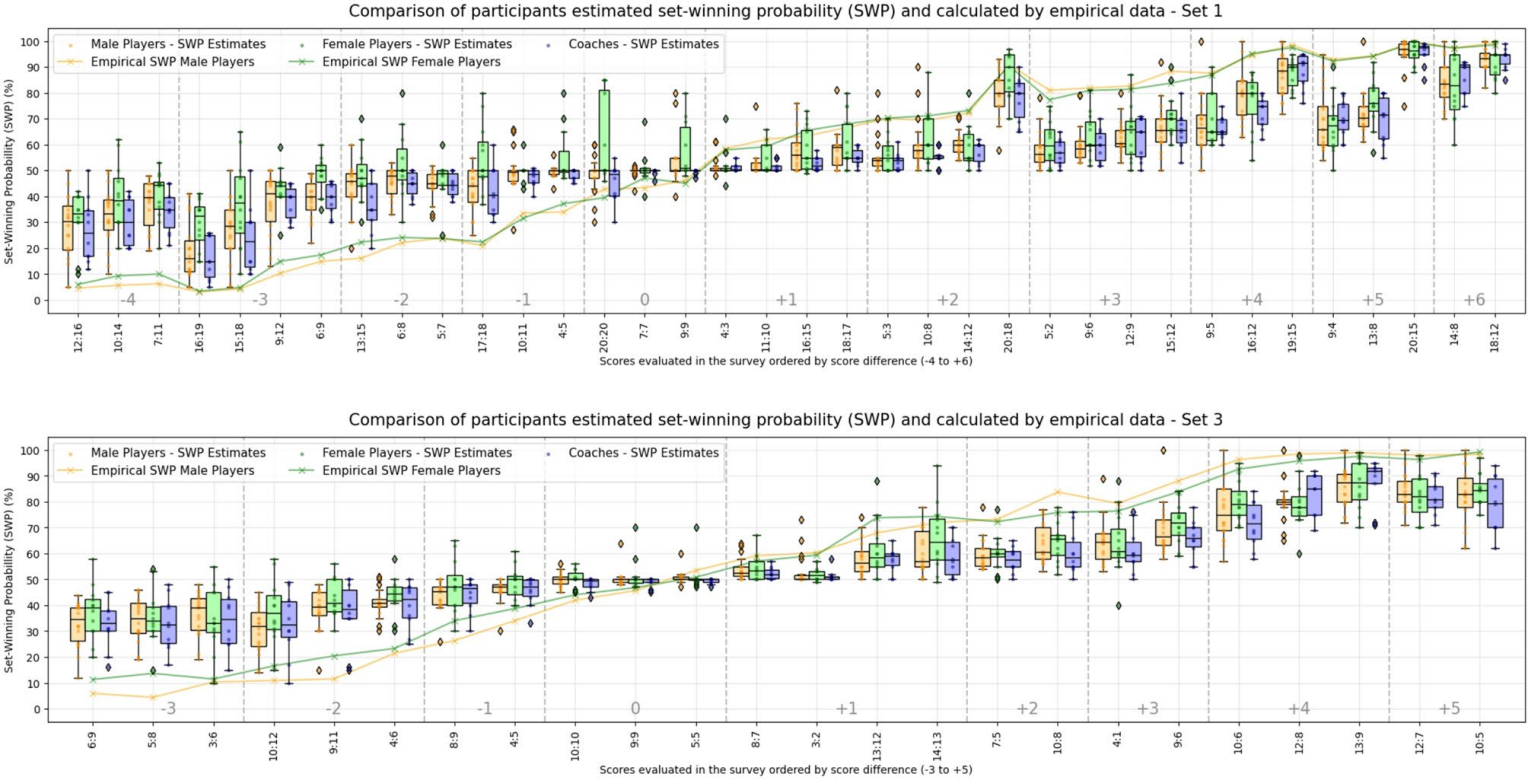
Participants estimated SWPs compared to empirical calculated SWPs for each evaluated score in the first set (top) and the third set (bottom) of the survey. Scores on the y-axis are ordered by the score difference of each score for better readability and do not represent the order in the survey. For coaches, both sexes are displayed together, as there is only one female coach in the sample.

To answer question 1 regarding the accuracy of experts estimating the SWP, Fig. 1 displays the SWPs estimated by the participants divided into participant groups alongside the empirically calculated SWP for each score in the survey. Results show that participants overestimated the SWP when trailing or in case of a tie and underestimated the SWP when leading. Notably, when leading and approaching the end of the set (e.g., within three points of winning), participants demonstrated significant smaller estimation discrepancies (*t* = 10.06; *p* < .001; *d* = 0.77) with SWP-ED_mean_ = -8% compared to other leading scores (SWP-ED_mean_ = -16%). Conversely, when trailing in this important phase (e.g., 16:19 or 15:18), participants exhibited substantial overestimation of the SWP (SWP-ED_mean_ = + 22%) with only slightly and not significant (*t* = 1.30; *p* = .20; *d* = 0.15) less discrepancy compared to other trailing scores (SWP-ED_mean_ = + 24%). Participants showed no substantial differences in SWP estimation precision between both sets.

Analyzing the SWP estimates for crucial situations, e.g., substantial trailing or tie, reveals some new information. First, in tied situations, participants consistently estimated the SWP close to 50% or higher, seemingly neglecting the specific match context – such as the disadvantage of serving and the need to score a breakpoint to take the lead. Second, some participants estimated the SWP in cases of substantial trailing higher than 50%, although the empirically calculated SWP was below 5–18%. Third, participants estimated the SWP for a 12:16 trailing score in the first set to be between 17% and 39%. However, when leading with the same score (16:12), the SWP was estimated to be between 66% and 86%, or 14–34% for the trailing team. Although reducing the score gap when trailing requires scoring a breakpoint, while in a leading scenario, the opponent can close the gap by scoring their side-out, which is a scenario with a higher probability than achieving a breakpoint. In summary, participants estimated their own chances of reducing a trailing score as higher than their opponent’s chances.

**Fig. 2:**
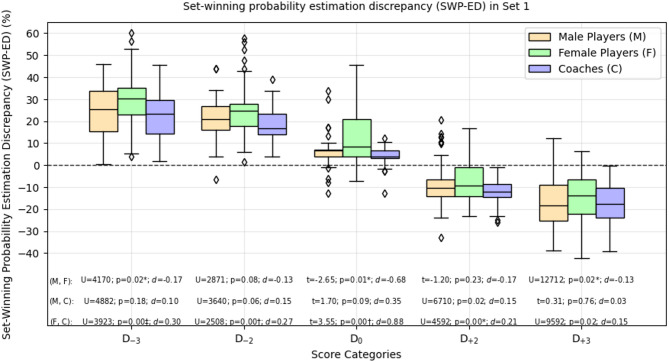
Set-winning probability estimation discrepancy (SWP-ED) of participants in the first set across five score categories. The red dotted line indicates perfect estimation without any discrepancy, whereas estimation above indicates over- and below underestimation of the SWP. The SWP-ED is calculated as the difference between a participant’s SWP estimate and the empirically calculated SWP for the corresponding score. Independent t-tests or Mann-Whitney U tests were conducted for group comparisons within each score category. To account for multiple comparisons, a Bonferroni correction was applied, adjusting the significance level to α / *m*, where *m* is the number of tests conducted per score category. Significant results are marked with asterisk (*, *p* < .05 / *m*), dagger (†, *p* < .01 / *m*), or double dagger (‡, *p* < .001 / *m*).

Figure 2 illustrates the differences in SWP-ED among the participant groups in the first set, highlighting the previously described patterns of over- and underestimation of the SWP across score categories. For participant estimates in the first set, the results indicate that female players’ estimates differed significantly from coaches’ estimates in four out of five score categories (*p* < .05 / 3 to *p* < .001 / 3) with moderate to high effect sizes (*d* = 0.21 to *d* = 0.88). In contrast, male players’ estimates showed no significant differences to coaches’ estimates. Additionally, significant differences were observed between female and male players’ estimates in the D_-3_ (*U* = 4170, *p* < .05 / 3, *d* = 0.17), D_0_ (*t* = 2.65, *p* < .05 / 3, *d* = 0.68), and D_+3_ (*U* = 12712, *p* < .05 / 3, *d* = 0.13) categories. In the third set, however, the pattern of significant differences was less pronounced (Supplementary Figure S2). Here, differences were observed only in D_+3_, with female players’ estimates differing from coaches’ estimates (*U* = 3592, *p* < .05 / 3, *d* = 0.24). No significant differences were detected between male players’ estimates and other groups’ estimates in this set.

### Which strategies do experts pursue when trailing or leading?

The analysis of the players’ and coaches’ responses was organized around four key questions that guided the identification of tactical patterns. 58% of players and 27% of coaches agreed that specific tactics should be used to maintain a lead of three or more points. The responses regarding the strategies used in this situation highlighted maintaining pressure, managing risks, and adapting strategies. One common theme was *maintaining pressure*, where players and coaches emphasized consistent pressure through strong serves and controlling the game’s rhythm. This included varying serve techniques, adjusting pace, and using tactics like time-outs to disrupt the opponent’s flow. *Risk management* emerged as another key theme. Participants discussed balancing aggressive play with strategic caution and adjusting serve risks based on the situation to maintain steadiness without unnecessary errors. For the theme *Adapting strategies based on the opponent’s play*, players and coaches highlighted the importance of flexibility, modifying tactics to exploit the opponent’s weaknesses or disrupt their rhythm.

The responses about what a large lead or high winning probability means to the team emphasized maintaining focus, lowering pressure, strategic confidence, and adaptability. The most common theme was *maintaining focus*. Players and coaches emphasized the need to stay concentrated on each point, avoiding complacency and ensuring consistent performance to protect the lead. The second theme, *reduced pressure*, highlights the sense of ease that comes with a lead. Players felt more freedom and confidence, with less anxiety about making mistakes, allowing them to execute their plays more comfortably. *Strategic confidence* emerged as a sign of assured performance. Participants saw a lead as confirmation of their strategy, boosting their confidence and control over the game. Notably, however, 6 out of 44 participants stated that a lead of 3 points or more holds no particular significance. They emphasized that the same level of focus should be maintained for every point.

85% of players and 64% of coaches agreed that specific strategies should be used to overcome a deficit of three or more points. The responses to which strategies are used in this situation focused on serve pressure, tactical adjustments, communication, and composure. One common strategy was increasing *serve pressure*. Players and coaches highlighted the importance of increasing the intensity and risk in their serves to disrupt the opponent’s rhythm and create break opportunities. Aggressive serving was seen as a way to force errors and gain momentum. *Tactical adjustments* emerged as another key theme. They emphasized the need to be flexible and adapt their tactics mid-game, making strategic changes to counter the opponent’s strengths. This included varying serves, adjusting defensive strategies, and finding new ways to exploit weaknesses. Lastly, *communication and composure* played a crucial role. Players and coaches underlined the need for clear team communication, effective use of time-outs, and staying calm under pressure. This allowed them to make thoughtful adjustments and maintain focus during critical moments.

The final guiding question 3, of what a high deficit or a low probability of winning meant for the team revealed a mix of psychological and tactical responses. The most common theme here was *tactical adjustments*: Players and coaches spoke about increasing the pressure and adjusting the defense strategies to make a comeback. They noted the importance of especially increasing the serve pressure, mentioning aspects of tactical adjustments in their answers. Additionally, the responses indicated that teams might adopt their *mental focus*, maintaining a positive mindset, resilience, and a focus on small goals to avoid feeling overwhelmed by the deficit. One athlete remarked, “You still have to give everything to turn the set or match around, focusing less on the score and more on a task-oriented approach to the next actions”. For a comprehensive list and detailed assignment of all answers and themes, we refer to the Supplementary Material.

### Do cognitive biases influence the estimations and encourage persistence in challenging situations in beach volleyball competition?

**Fig. 3:**
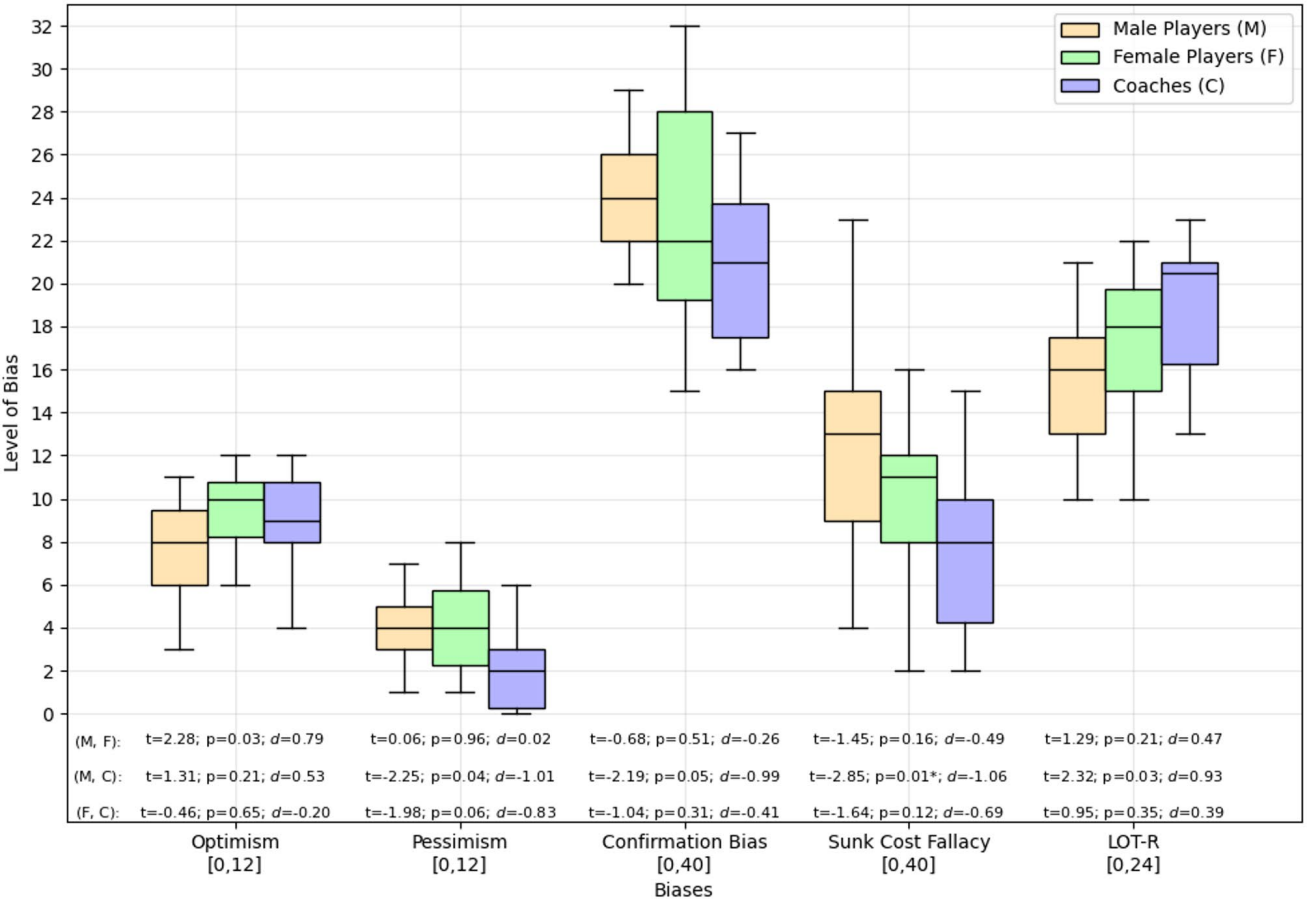
Distribution of participants’ responses to the assessed bias questionnaires. The range of possible minimum and maximum scores for each bias is indicated below the x-axis labels. Independent t-tests were conducted for group comparisons within each bias. To account for multiple comparisons, a Bonferroni correction was applied, adjusting the significance level to *p* = .05 / *m*, where *m* is the number of tests conducted per score category. Significant results are marked with an asterisk (*p* < .05).

In the survey, participants completed three different validated questionnaires to assess their levels of optimism and pessimism, susceptibility to the sunk cost fallacy, and confirmation bias. Figure 3 displays the participants’ responses, including the LOT-R value, as the combined score of optimism and pessimism, along with additional information on whether the participant groups differed in their answers. Results indicate only one significant difference between the groups and across the five assessed measurements, whereas the sunk cost fallacy answers of coaches and male players differed significantly (*t* = 2.85; *p* = .01; *d* = 1.06). Although there was only one significant difference, the distribution of biases across the groups showed moderate differences and high effect sizes, e.g., optimism-mean_male_players_ = 7.8 and optimism-mean_female_players_ = 9.4, indicating group-specific characteristics. More detailed information on the distribution of biases can be found in Supplementary Table S1.

To assess those variables that influence the level of under- and overestimation in the defined score categories, we trained linear regression models for each combination of score category, participant group, and influencing variable. Table 2 shows the Pearson correlation results of all single linear regressions. Additionally, figures for each linear regression are given in the Supplementary Material. Interestingly, in case of trailing (D_-2_ and D_-3_), four out of five tested variables show significant correlations among the participant groups with moderate to weak relationships^50^, but not for the sunk cost fallacy answers. For leading scores, fewer variables show significant relationships. Conversely, in case of tie scores, no significant relationship could be seen. The optimism values show significant correlations among all three participant groups. The confirmation bias has for trailing and leading scenarios positive correlations for female players indicating a high relationship to their answer behavior, though the correlation is lower in case of leading. Vice versa, male players showed no significant correlation for the confirmation bias among any score category. Pessimism negatively correlates with the SWP estimates indicating that lower pessimism comes with higher overestimation (trailing) and lower underestimation (leading) which is the opposite of the optimism results. Note, for optimism, an exception occurs where the correlation is negative with coaches’ estimates and leading scenarios indicating that coaches with higher optimism underestimated the SWP more. Supplementary Table S2 reports the single linear regression results for the full sample; here, optimism, confirmation bias, and the LOT-R also showed significant results in trailing scenarios, consistent with the findings above.

Subsequently, we trained 15 robust linear models (RLM) to evaluate the explained variance (Pseudo-*R*^2^) of the assessed decision-making tendencies across all participant groups and score categories. These regressions included all potentially influencing variables: optimism, pessimism, sunk cost fallacy, and the confirmation bias. The results, summarized in Table 3, highlight the RLMs with significant *β*-coefficients for an influencing variable. Overall, the analyses revealed low Pseudo-*R*^2^ values across the RLMs. The highest Pseudo-*R*^2^ was observed among female players in D_-3_ with Pseudo-*R*^2^ = 0.313 and a strong effect size of *f*^2^ = 0.456^50^, where the confirmation bias had a highly significant positive effect (*β* = 1.21, 95% CI [0.77, 1.66]). Additionally, moderate effect sizes were yielded by female players in D_−2_ and D_3_ and by coaches in D_-3_ and D_-2_ (0.15 < *f*^2^ < 0.35). Unlike the single linear regression analyses, optimism had only a minor impact on the RLM results. In contrast, confirmation bias emerged as a strong influencing factor across all tested groups, with its most pronounced effects in D_-2_ and D_-3_. Among male players, the RLMs indicated highly significant negative effects of pessimism, particularly in substantial trailing score scenarios. Supplementary Table S3 reports the RLM results for the full sample, showing significant *β*-coefficients for optimism and confirmation bias in trailing scenarios but with low Pseudo-*R*^2^ (0.089 to 0.110) and small effect sizes (0.097 to 0.124). A complete overview of all RLM results is provided in the Supplementary Material.

**Table 2:** Pearson correlation (*r*) results of linear regression models for each participant group (M, F, C), score category, and assessed decision-making tendency as independent variable.

	Optimism			Pessimism			Confirmation Bias		
	M	F	C	M	F	C	M	F	C
D_-3_	0.17*	**0.32‡**	**0.30†**	**− 0.28‡**	**− 0.31‡**	− 0.07	− 0.07	**0.46‡**	**0.35‡**
D_-2_	**0.20†**	0.12	**0.43‡**	**− 0.24‡**	− 0.10	− 0.10	− 0.09	**0.27†**	**0.39‡**
D_0_	0.16	0.08	0.22	− 0.17	− 0.05	0.10	0.01	0.10	0.23
D_+2_	0.06	**0.23†**	− 0.09	− 0.14*	− 0.09	− 0.16	0.05	0.18*	− 0.04
D_+3_	0.00	**0.25‡**	**− 0.21†**	− 0.01	− 0.13	− 0.00	0.10	**0.19†**	− 0.10

	Sunk Cost Fallacy			LOT-R		
	M	F	C	M	F	C
D_-3_	− 0.03	0.15	− 0.00	**0.25‡**	**0.33‡**	**0.26†**
D_-2_	0.01	− 0.03	0.10	**0.25‡**	0.11	**0.37‡**
D_0_	0.02	− 0.05	0.13	0.19	0.07	0.09
D_+2_	0.14*	− 0.01	0.09	0.11	0.16*	0.04
D_+3_	0.09	− 0.01	0.07	0.01	**0.19†**	− 0.15*

In bold, an asterisk (*, *p* < .05), dagger (†, *p* < .01), or double dagger (‡, *p* < .001) indicate significant Pearson correlations. For trailing scenarios (D_-3_ and D_-2_), a negative correlation indicates that higher values of the independent variable led to better estimates, as participants tended to overestimate the SWP. For leading scenarios (D_+2_ and D_+3_),a negative correlation suggests more estimation discrepancy or higher underestimation if the independent variable is higher, as participants in our survey predominantly underestimated the SWP.

**Table 3:** Results of robust linear models across participant groups (M, F, C) and score categories with the four assessed decision-making tendencies as independent variables.

		Pseudo- *R* ^2^	Cohen’s *f* ^2^	Optimism	Pessimism	Confirmation Bias	Sunk Cost Fallacy
D_-3_	*F*	*0.313*	*0.456*	*1.67*	*-0.14*	***1.21‡***	*-0.16*
				*[–0.64; 3.97]*	*[–2.43; 2.16]*	*[0.77; 1.66]*	*[–0.80; 0.48]*
	*C*	*0.211*	*0.268*	*0.56*	*-0.05*	***1.55‡***	***-0.96†***
				*[–0.33; 1.45]*	*[–0.89; 0.79]*	*[0.80; 2.29]*	*[–1.60; − 0.32]*
	*M*	*0.090*	*0.098*	*0.02*	***-2.36†***	*0.09*	*-0.30*
				*[–1.05; 1.08]*	*[–3.94; − 0.79]*	*[–0.80; 0.97]*	*[–0.82; 0.22]*
D_-2_	*F*	*0.162*	*0.194*	*0.29*	*-0.69*	***1.07‡***	-0.68*
				*[–2.13; 2.72]*	*[–3.10; 1.72]*	*[0.59; 1.55]*	*[–1.35; − 0.01]*
	*C*	*0.221*	*0.284*	0.93*	*-0.17*	***0.97†***	*-0.51*
				*[0.18; 1.69]*	*[–0.89; 0.54]*	*[0.33; 1.60]*	*[–1.06; 0.03]*
D_2_	*F*	*0.119*	*0.135*	***3.50‡***	***2.78†***	0.37*	*0.27*
				*[1.83; 5.18]*	*[1.11; 4.44]*	*[0.04; 0.70]*	*[–0.19; 0.74]*
	*M*	*0.027*	*0.028*	*0.49*	*0.29*	*-0.19*	0.34*
				*[–0.06; 1.04]*	*[–0.52; 1.11]*	*[–0.65; 0.26]*	*[0.07; 0.61]*
D_3_	*F*	*0.136*	*0.158*	***3.44‡***	***2.40†***	***0.49†***	*0.20*
				*[1.79; 5.08]*	*[0.76; 4.04]*	*[0.17; 0.82]*	*[–0.26; 0.65]*
	*C*	*0.062*	*0.066*	-0.86*	*0.05*	*-0.25*	*0.44*
				*[–1.62; − 0.09]*	*[–0.68; 0.77]*	*[–0.89; 0.40]*	*[–0.12; 0.99]*

The confidence interval [0.025, 0.975] is shown in brackets below the *β*-coefficient-values. Only those results with at minimum one significant *β*-coefficient are listed. In bold and with an asterisk (*, *p* < .05),dagger (†, *p* < .01), or double dagger (‡, *p* < .001) indicate significant *β*-coefficients. Cohen’s *f*^2^ values represent effect size: small (≥ 0.02), medium (≥ 0.15), and large (≥ 0.35) according to Cohen^50^.

### Integration of SWP estimations and strategic responses

An integrated analysis of SWP estimations and reported strategies reveals distinct patterns in how participants perceive and respond to different game situations. In substantial leading scenarios (D_+3_), participants tended to underestimate the SWP. Correspondingly, their reported strategies emphasized risk management and maintaining control, with a focus on consistent play and minimizing errors to secure their lead. Conversely, when substantial trailing (D_-3_), participants consistently overestimated the SWP. In these situations, they reported employing strategies such as increasing serve pressure and making tactical adjustments to disrupt the opponent’s rhythm and create break opportunities. Further analyses indicate that biases had a stronger influence on participants’ SWP estimates in substantial leading (D_+3_) and trailing (D_−3_) scenarios compared to moderate scenarios (D_−2_ or D_+2_) or ties (D_0_). This suggests that the degree of point difference intensifies the impact of cognitive biases on participants’ estimates and their reported strategic decisions and indicates the added value of our mixed-methods approach to enhance athletes’ performance.

## Discussion

In the current study, we aimed to shed light on how elite beach volleyball players and coaches estimate set-winning probabilities (SWPs) in various game scenarios. Our findings revealed a consistent pattern: SWPs were generally underestimated when leading and overestimated in the case of trailing and a tie. These discrepancies highlight a potential cognitive bias in perceiving such situations that may influence decision making during critical moments in competition.

When trailing, players and coaches reported adopting high-risk strategies, such as increasing serve pressure and implementing tactical adjustments. This indicates an awareness of the need for significant strategic changes to alter the game’s trajectory. However, when these strategies are considered alongside the observed overestimation of SWPs in trailing scenarios, it becomes evident that players may not fully comprehend the severity of their disadvantage. This is supported by the observation that 15% of players and 36% of coaches did not agree to apply a specific strategy in case of substantial trailing (with 3 or more points). However, in such cases the probability of winning the set falls below 20%.

Overestimating SWPs in case of trailing could lead to underestimating the urgency for immediate strategic adjustments, potentially delaying critical decisions. Our findings tentatively suggest that optimism may involve both state-like and trait-like components in this context^23^. State-like optimism may help explain fluctuations in estimates depending on situational factors, such as whether participants are trailing or leading. At the same time, there was also evidence suggesting trait-like tendencies, as participants with higher general levels of optimism tended to provide higher SWP estimates across scenarios. These findings tentatively support the notion that optimism may function both as a situationally influenced process and as a relatively stable individual difference. An important implication for competitive practice is that tactical adjustments may occur too late^8^, as coaches and players struggle to accurately identify the critical point at which winning probabilities drop into a precarious range. However, we also acknowledge that simply presenting low empirical SWPs to players may seem discouraging or reduce effort. Our intention is not to undermine motivation, but to increase situational awareness. Understanding the severity of a trailing situation can help trigger necessary tactical adjustments. When communicated within a supportive and reflective training context, such insights can enhance decision making without diminishing players’ persistence or belief in a possible comeback. Strategies commonly proposed by coaches, which align with the players’ perspectives, include drastically increasing serve pressure or adopting more aggressive defensive tactics. One unique aspect of beach volleyball is that the coach is not allowed to intervene or provide support during the match. Therefore, it becomes even more essential for the players to develop their own game strategies and accurately assess game situations. It is crucial to recognize when the current strategy is no longer effective or is being poorly executed and to promptly shift to a higher-risk alternative. Without such a tactical adjustment, the statistical probability of winning becomes increasingly unfavorable.

In conclusion, for trailing scenarios, our analysis showed that optimism and the confirmation bias significantly influenced players’ SWP overestimations. Nickerson^51^ argued that confirmation bias, as the “seeking or interpreting of evidence in ways that are partial to existing beliefs” may produce performance detriments. Thus, the bias can lead to systematic errors in judgment and decision making, influencing how individuals process and recall information. Our findings suggest that players are more likely to recall past sets where a team overcame a 4-point deficit to win. However, empirical data indicate that only 6 to 10 matches out of 100 were won in such situations. The selective recognition of these rare events, combined with high optimism, fosters the belief, “We can still win”. This tendency may be further reinforced by the cognitive salience of rare comebacks, as described in research on availability heuristics (e.g., Tversky & Kahneman^52^. Interestingly, players with higher levels of pessimism demonstrated more accurate estimates when trailing, suggesting they may benefit from their more conservative outlook.

Conversely, when leading, participants emphasized strategies focused on risk management and maintaining continuity, such as minimizing errors and maintaining focus. These cautious approaches align with their underestimation of SWPs, reflecting a perception of precariousness even when in a strong position. While this approach can mitigate unnecessary risks, it may also suggest a tendency toward conservatism that could allow opponents to regain momentum. This behavior could be influenced by a pessimism bias, as the results show that greater underestimation of SWPs is associated with higher levels of pessimism among both male and female players. As in trailing scenarios, confirmation bias also correlates with SWP estimates in leading scenarios. However, in this case, higher levels of confirmation bias do not lead to greater estimation discrepancies (in this case underestimation); instead, they are associated with more accurate estimates. This is particularly significant for female players, who seem to recall sets more accurately where a lead resulted in victory. These findings suggest that confirmation bias functions differently depending on the context: in trailing scenarios, it amplifies SWP overestimation, leading players to overlook the significant disadvantage they face. In contrast, in leading scenarios, it enhances estimation accuracy by concentrating on the victory. Both observations, confirmation bias leading to greater SWP-ED when trailing but reducing it when leading, support an overly optimistic perspective on the game. Participants are more likely to remember scenarios that reinforce the belief, “We (can still) win”.

Notably, players seem to demonstrate self-confidence in their abilities, regardless of whether they were leading or trailing. For example, when trailing by four points, participants estimated their SWP at 17–39%, while they estimated it at 66–86% when leading by the same margin. Ideally, one would expect an inverse relationship between these probabilities, but with a small disadvantage for the leading scenario as the leading team serves. Yet, participants rated the leading scenario higher than the trailing situation. Additionally, the consistent overestimation in tie situations could be caused by self-confidence. This upward bias reflects an optimistic belief in their ability to recover from deficits or maintain advantages, which may stem from a general confidence in their performance. Self-confidence is widely recognized as a critical factor in sports performance, with studies indicating that higher levels of self-confidence are linked to improved outcomes^53,54^. Self-confident athletes are more likely to persist through challenges, execute their skills effectively, and maintain composure under pressure – qualities essential for success in elite sports.

The findings of this study offer practical insights for improving decision- making and strategy in elite beach volleyball. Training programs should prioritize enhancing players’ ability to accurately assess game situations, particularly addressing the overestimation of winning probabilities when trailing, as this is often critical for turning a match around. Incorporating match data into training can help players recognize the urgency of timely tactical adjustments. Besides that, through targeted psychoeducation, players can be made aware of the cognitive biases influencing their decision making. By understanding these biases, athletes can better recognize and potentially mitigate their impact, leading to more informed and strategic choices during competition.

Although our study provides valuable insights into the perceptions of winning probabilities in beach volleyball, several limitations should be considered. First, the study focused exclusively on elite beach volleyball players and coaches, which may limit the applicability of the findings to other skill levels or sports. Second, all participants were from Germany. This homogeneity may have influenced the assessment of winning probabilities and cognitive biases, as cultural background can significantly shape such evaluations. Third, the low number of female coaches in the survey could limit the generalizability of the findings, as they might offer different perspectives compared to male coaches. Fourth, participants frequently estimated SWPs around 50%, which could reflect a bias toward mean values. Fifth, the linear regression and robust linear model analyses yielded low Pearson correlations and low to moderate explained variances, with only moderate effect sizes. This indicates that our study could not comprehensively explain the phenomenon under investigation. Sixth, another possible explanation for the observed estimation patterns is the influence of the availability heuristic. Players may overestimate their chances when trailing because they vividly recall rare but memorable comebacks, while underestimating SWP when leading could stem from salient memories of lost leads. Although this mechanism may partly overlap with confirmation bias, we did not explicitly assess the types or frequency of recalled situations in our study.

Future research could explore strategies to counteract these biases or investigate whether it might serve a functional purpose in team sports. For instance, studies could examine whether athletes with a stronger confirmation bias are more likely to overcome deficits compared to those with a less pronounced bias. Given that the examined biases account for only a limited portion of the observed estimation patterns, future research should broaden the scope by investigating additional cognitive mechanisms that may better explain variance in SWP estimations.

## Conclusion

This study highlights the influence of cognitive biases on set-winning probability estimates and strategic decision making in elite beach volleyball. Players and coaches tend to perceive the game as more even than it actually is, leading to systematic over- and underestimations that may delay crucial tactical adjustments. These estimates might be influenced by the optimism and confirmation bias. Addressing these biases through targeted training and psychoeducation can enhance players’ situational awareness and decision making, ultimately improving their competitive performance in beach volleyball.

## Supplementary Information

Below is the link to the electronic supplementary material.

Supplementary Material 1

## Data Availability

The datasets generated and/or analyzed during the current study are not publicly available due to the authors being not the owner of all data, but they are available from the corresponding author on reasonable request.
