# Supplementary material for: How cognitive biases affect winning probability perception in beach volleyball experts
Source: Sci Rep. 2025 Sep 11;15:32408. doi: 10.1038/s41598-025-17770-z (PMC12426222; doi:10.1038/s41598-025-17770-z)
Supplement: Supplementary file 1 — Supplementary Material 1 [file 41598_2025_17770_MOESM1_ESM.pdf]

# Supplementary Material


1. Additional Figures and Tables referenced in the Manuscript ........ 2
2. Data processing ........ 4
   a. Utilized approach: customized outlier handling ........ 5
   b. Alternative data processing approach: winsorizing ........ 5
3. Comprehensive tables of all answers for qualitative questionnaire questions ........ 5
4. Tables and Figures of regression analyses ........ 10
   a. Figures of all single linear regression models ........ 10
   b. Complete results of robust linear models ........ 14
5. Results for alternative outlier handling ........ 18
   a. Winsorizing approach ........ 19
   b. No outlier handling ........ 23
6. Comparison of linear regression results with different outlier handling approaches ........ 27

# 1. Additional Figures and Tables referenced in the Manuscript

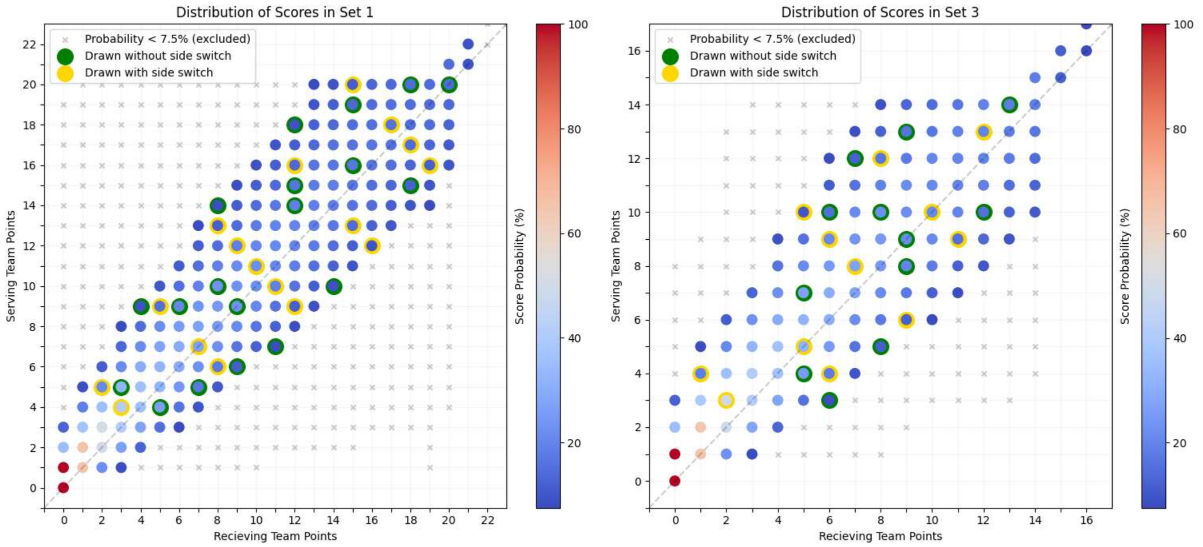


*Figure S1: Distribution and selection of the scores for the first set (left) and the third set (right). Yellow-bordered points represent the drawn scores from all side switches, while green-bordered points indicate the drawn scores from all remaining scores.*

*Table S1: Participants bias levels measured by the questionnaires. Shown are the mean and standard deviation within the group.*

| | **Players** | | | | | | | | | **Coaches** | | |
|---|---|---|---|---|---|---|---|---|---|---|---|---|
| **Sex** | M (n=19) | | | F (n=14) | | | All (n=33) | | | All (n=10) | | |
| | Mean ± SD | min | max | Mean ± SD | min | max | Mean ± SD | min | max | Mean ± SD | min | max |
| **LOT-R [0-24]** | 15.7 ± 3.1 | 10 | 21 | 17.3 ± 3.8 | 10 | 22 | 16.4 ± 3.5 | 10 | 22 | 18.7 ± 3.4 | 13 | 23 |
| **Optimism [0-12]** | 7.8 ± 2.2 | 3 | 11 | 9.4 ± 1.9 | 6 | 12 | 8.5 ± 2.2 | 3 | 12 | 9.0 ± 2.4 | 4 | 12 |
| **Pessimism [0-12]** | 4.1 ± 1.4 | 1 | 7 | 4.1 ± 2.1 | 1 | 8 | 4.1 ± 1.7 | 1 | 8 | 2.3 ± 2.3 | 0 | 6 |
| **Confirmation [0-40]** | 24.1 ± 2.5 | 20 | 29 | 23.0 ± 5.4 | 15 | 32 | 23.6 ± 4.0 | 15 | 32 | 21.0 ± 4.0 | 16 | 27 |
| **Sunk Cost [0-40]** | 12.6 ± 4.9 | 4 | 23 | 10.4 ± 3.8 | 2 | 16 | 11.6 ± 4.6 | 2 | 23 | 7.6 ± 4.2 | 2 | 15 |

*Note. Coaches are not split by sex, as only one female coach was part of the study. LOT-R is the combination of optimism and pessimism and the result of the Life Orientation Test-Revised [36].*

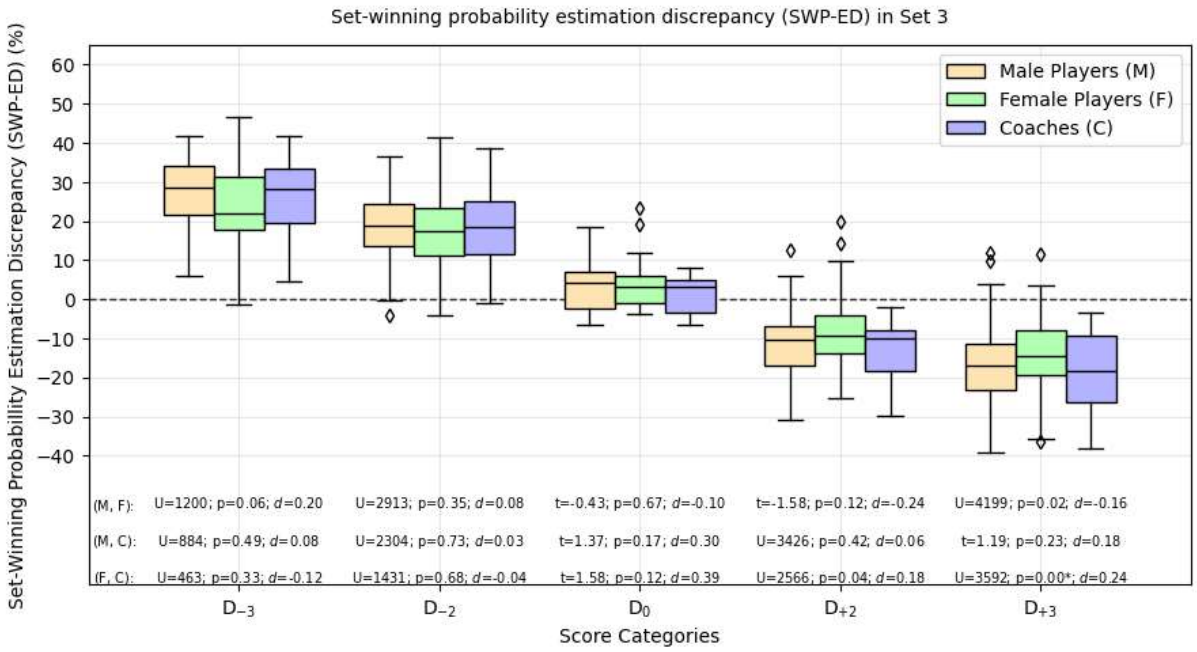


*Figure S2: Participants set-winning probability estimation discrepancy (SWP-ED) in the third set across five score categories. The red dotted line indicates perfect estimation without any discrepancy, whereas estimation above indicates over- and below underestimation of the SWP. The SWP-ED is calculated as the difference between a participant's SWP estimate and the empirically calculated SWP for the corresponding score. Independent t-tests or Mann-Whitney U tests were conducted for group comparisons within each score category. To account for multiple comparisons, a Bonferroni correction was applied, adjusting the significance level to α / m, where m is the number of tests conducted per score category. Significant results are marked with asterisk (*, p < .05 / m), dagger (†, p < .01 / m), or double dagger (‡, p < .001 / m).*

For transparency, we provide in Table S2 the Pearson correlation results of linear regression models for the full sample and without participant group split. Complete results and visualization of regression lines are given in Figure S8.

*Table S2: Pearson correlation (r) results of Linear Regression models for each score category and assessed decision-making tendency as independent variable.*

| | Optimism | Pessimism | Confirmation Bias | Sunk Cost Fallacy | LOT-R |
|---|---|---|---|---|---|
| $D_{-3}$ | **.24‡** | **-.16†** | **.23‡** | .04 | **.24‡** |
| $D_{-2}$ | **.22‡** | -.09 | **.19‡** | .04 | **.19‡** |
| $D_0$ | **.14*** | .03 | .08 | .03 | .07 |
| $D_{+2}$ | .07 | -.04 | **.09*** | **.11†** | .07 |
| $D_{+3}$ | .03 | -.00 | .06 | .05 | .02 |

*Note: In bold, an asterisk (*, p < .05), dagger (†, p < .01), or double dagger (‡, p < .001) indicate significant Pearson correlations. For trailing scenarios ($D_{-3}$ and $D_{-2}$), a negative correlation indicates that higher values of the independent variable led to better estimates, as participants tended to overestimate the SWP. For leading scenarios ($D_{+2}$ and $D_{+3}$), a negative correlation suggests more estimation discrepancy or higher underestimation if the independent variable is higher, as participants in our survey predominantly underestimated the SWP.*

Table S3 shows the results of robust linear models for the full sample across score categories with the four assessed decision-making tendencies as independent variables. Complete results of all trained RLMs are given in Table S9.

*Table S3: Results of robust linear models on the full sample across score categories with the four assessed decision-making tendencies as independent variables.*

| | Pseudo-$R^2$ | Cohen's $f^2$ | Optimism | Pessimism | Confirmation Bias | Sunk Cost Fallacy |
|---|---|---|---|---|---|---|
| $D_{-3}$ | .110 | .124 | **1.10 ‡** | -0.21 | **0.78 ‡** | -0.07 |
| | | | [0.53; 1.67] | [-0.81; 0.39] | [0.43; 1.14] | [-0.36; 0.21] |
| $D_{-2}$ | .089 | .097 | **0.84 ‡** | -0.03 | **0.57 ‡** | 0.02 |
| | | | [0.38; 1.31] | [-0.52; 0.46] | [0.27; 0.86] | [-0.21; 0.25] |
| $D_0$ | .026 | .027 | 0.30 | **0.41*** | 0.17 | 0.10 |
| | | | [-0.08; 0.68] | [0.01; 0.82] | [-0.07; 0.41] | [-0.09; 0.29] |
| $D_{+2}$ | .022 | .022 | 0.30 | 0.04 | 0.00 | 0.17 |
| | | | [-0.04; 0.64] | [-0.32; 0.40] | [-0.21; 0.22] | [0.00; 0.34] |
| $D_{+3}$ | .006 | .006 | 0.18 | 0.03 | 0.09 | 0.07 |
| | | | [-0.23; 0.60] | [-0.41; 0.46] | [-0.17; 0.35] | [-0.13; 0.28] |

*Note: The confidence interval [0.025, 0.975] is shown in brackets below the β-coefficient-values. In bold and with an asterisk (*, $p < .05$), dagger (†, $p < .01$), or double dagger (‡, $p < .001$) indicate significant β-coefficients. Cohen's $f^2$ values represent effect size: small (≥ 0.02), medium (≥ 0.15), and large (≥ 0.35) according to Cohen [50].*

## 2. Data processing

In Figure S3 the distribution of each participant's raw answers in our survey is provided. For comparison, in Figure S4, the result after step 1 is shown.

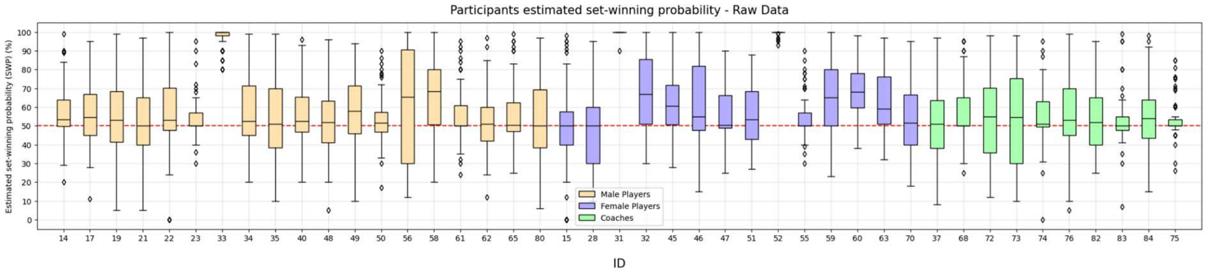


*Figure S3: Participants estimated set-winning probability - Raw Data*

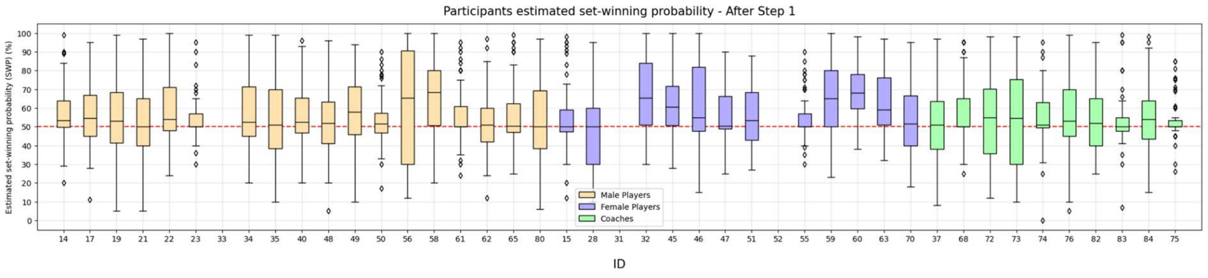


*Figure S4: Participants estimated set-winning probability after step 1.*

## a. Utilized approach: customized outlier handling

As a result of our customized outlier handling, we show in Figure S5 the estimated set-winning probabilities after described and applied step two.

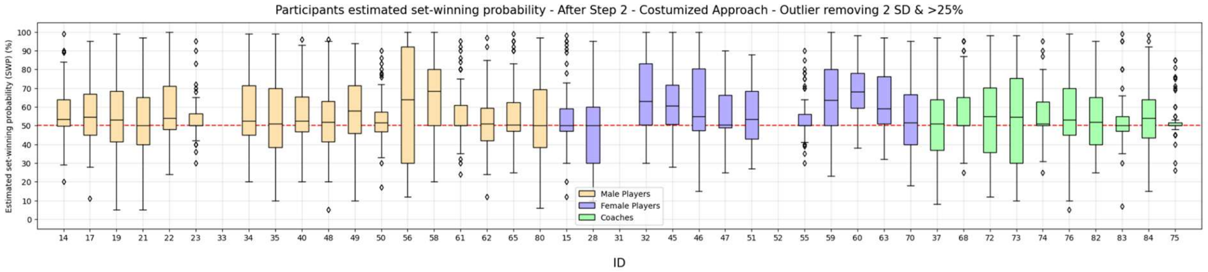


*Figure S5: Participants estimated set-winning probability after step 2 with our utilized customized approach. Outlier estimates that differed by more than two standard deviations from the sample mean and that deviated by more than 25% above or below the empirical SWP were excluded.*

## b. Alternative data processing approach: winsorizing

As an alternative, we utilized a standardized winsorizing approach. In this method, all outliers exceeding two standard deviations from the sample mean were replaced with the corresponding boundary value of two standard deviations (Fig. S6).

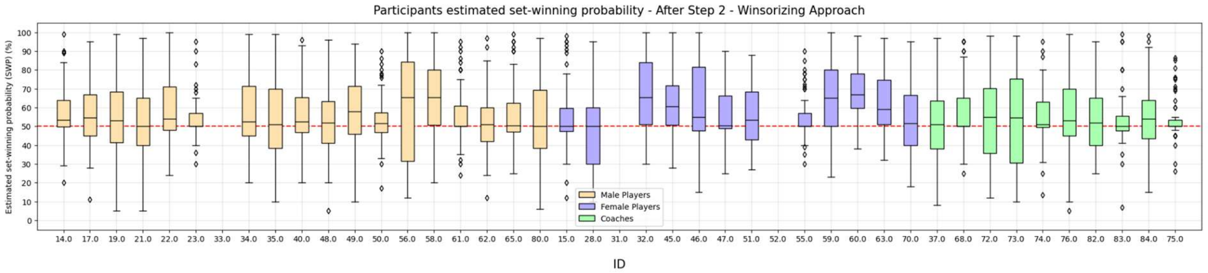


*Figure S6: Participants estimated set-winning probability after step 2 with an alternative winsorizing approach. All outliers exceeding two standard deviations from the sample mean were replaced with the corresponding boundary value of two standard deviations*

# 3. Comprehensive tables of all answers for qualitative questionnaire questions

On the following four pages, tables show the answers of all participants to the assessed qualitative questionnaire described in the manuscript. The original answers shown were assed in German and not edited but additionally translated to English. Table S4 shows answers to question 1, Table S5 to question 2, Table S6 to question 3, and Table S7 to question 4.

*Table S4: Comprehensive list and detailed assignment of all answers and themes to question 1*

| Question 1 | What strategies are used to maintain a lead of 3 or more points? | What strategies are used to maintain a lead of 3 or more points? | Maintaining Pressure | Risk management | Adapting strategies based on the opponent's play | Others |
|---|---|---|---|---|---|---|
| | **Original (GERMAN)** | **translated to English** | | | | |
| Athletes | Voller Fokus aufs Sideout. Im Aufschlag bewusst Risiko beibehalten und nicht dazu tendieren nur Fehler zu vermeiden | Full focus on side-out. Consciously maintain serve risk and avoid just playing safe to prevent errors | | x | | |
| | Fokus auf Taktik | Focus on tactics | | | x | |
| | weiter im Austausch mit der Partnerin bleiben (Taktikabsprache) | Stay in communication with partner (tactical coordination) | | | x | |
| | -mehr Fokus auf den Aufschlag - nach zwei nicht gemachten side-outs Seite wechseln oder Time-out nehmen | More focus on the serve – after two missed side-outs, switch sides or take a timeout | x | | | |
| | Weiterhin Aufschlagdruck, den Rhythmus des Spiels bestimmen (schnell/langsam -> was dem Gegner weniger liegt) | Maintain serve pressure, control the rhythm of the game (fast/slow depending on what the opponent dislikes) | x | | x | |
| | Aufschlagdruck | Serve pressure | x | | | |
| | Spielrhytmus beeinflussen, Brille putzen/ Linien richten etc. um das Tempo zu steuern und Kontrolle zu halten | Influence game rhythm, clean glasses/adjust lines to control pace and maintain control | | | | x |
| | Dann werden die vorher besprochenen Taktiken genutzt für die Bälle die von dem Gegner die Lieblingsbälle darstellen, da er jetzt vielleicht in sein Muster fällt. Und wenn ich zwei Sideouts nicht mache wird eine Auszeit genommen wenn diese noch vorhanden ist um den Gegner in seinem Rhythmus den er in dem Moment aufbaut zu stören. | Then use previously discussed tactics for the opponent's favorite shots as they may fall into patterns now... Take a timeout if two side-outs are missed to break their rhythm | | | x | |
| | Man geht nicht unnötiges Risiko ein, da man die Gegner sowieso ohne Probleme und oft genug breakt. | Avoid unnecessary risk, since the opponent is regularly broken without issue | | x | | |
| | sobald ein side out (je nach Situation auch 2) nicht erfolgreich ist, eine Auszeit nehmen | If one (or sometimes two) side-outs fail, take a timeout | | | | |
| | Druck auf Spielerin ausüben, die für Fehler bzw. unseren Vorsprung verantwortlich ist | Put pressure on the player responsible for errors or our lead | x | | | |
| | Jeden Ball aufs neue Fokussieren | Focus on every ball anew | | | | x |
| | Im Aufschlag nicht auf 80% Risiko gehen. Standard Situationen des Gegners präsent haben und diese Bälle abwehren wollen. | Don't serve with 80% risk. Be aware of opponent's standard patterns and defend those | | x | x | |
| | Man gibt dem Gegner ein anderes Bild, sei es im Aufschlag oder Block-Defense. Damit der Gegner sich nicht an eine Taktik gewöhnen kann. | Give the opponent a different look, whether through serve or block-defense, so they can't get used to one tactic | | | x | |
| | Die Spielstrategie, die bis zu diesem Zeitpunkt auch geholfen hat die Führung zu erspielen. | Stick to the game strategy that helped gain the lead up to this point | | | x | |
| | Kommt auf den Gegner an. Wenn beide Teams gutes Sideout spielen würde ich bei einer Führung viel Aufschlagrisiko gehen. Wenn beide Teams schlechtes Sideout spielen würde ich wenig Aufschlagrisiko gehen. | Depends on the opponent. If both teams have strong side-out, go high-risk. If both struggle with side-out, reduce serve risk | x | x | x | |
| | Auf eine Spielerin zu spielen; die Blockfeldabwehr ähnlich zu gestalten und den Rythmus beibehalten. | Target one player; keep block-defense similar and maintain rhythm | x | | | |
| | Weiterhin auf den schwächeren Side out Spieler aufschlagen. Ggf dem anderen Spieler an der anspielen für einen Rhythmuswechsel. Aufschlagrisiko konstant halten. ggf. eher platziert statt Risikoreich | Keep serving to the weaker side-out player. Occasionally serve the other one for rhythm disruption. Maintain consistent serve risk, possibly aim instead of powering | x | | | |
| | Fokus auf sideout | Focus on side-out | | | | |
| Coaches | zeitweise erhöhter Aufschlagdruck durch beide Aufschläger = Gegner soll nicht ins Spiel kommen bspw. im Voraus für 2./3. Satz erhöhter Aufschlagdruck durch den besseren Aufschläger 1 und weiterhin Taktikaufschlag durch Aufschläger 2, um Gegner unter Druck zu halten (bspw. wenn Zugriff über Block Abwehr in den vorangegangenen Ballwechseln sehr erfolgreich war) Auszeit nach bereits einem eigenen Fehler Spielbeschleunigung, d.h. zwischen den Ballwechseln wird ggf. der Rhythmus verkürzt, um Gegner dauerhaft zu beschäftigen Analyse, in welchem Bereich Gegner aktuell besonders unterlegen ist (Annahme, Angriff aus In/Off-System) | Temporary increased serve pressure by both servers = prevent opponent from getting into the game (e.g., from 2nd/3rd set onward)... analyze where the opponent is currently most vulnerable (reception, attack, etc.) | x | | x | |
| | Abhängigi von weiteren Faktoren: Mehr Risiko im Aufschlag Den Partner anspielen, um auf den eigentlichen Spieler am Ende zu wechseln Einen Spieler fertig machen (Hitze) | Depends on other factors: more serve risk, target the partner to switch to the main player later, wear down one player (e.g., due to heat) | x | | | |

*Table S5: Comprehensive list and detailed assignment of all answers and themes to question 2*

| Question 2 | What does a high lead or high win probability mean for you or your team? | | Maintaining focus | Reduced Pressure | Strategic Confidence | Others |
|---|---|---|---|---|---|---|
| | Original (GERMAN) | translated to English | | | | |
| Athletes | Komfortable Ausgangssituation. Jedoch ist ein Spannungsabfall zu verhindern. Also aufs wesentliche (Sideout) Konzentrieren um Führung zu halten. | Comfortable situation. However, avoid losing tension. Focus on essentials (side-out) to maintain the lead | x | x | x | |
| | Weniger Druck, Selbstbewusstsein, Möglichkeit freier zu spielen | Less pressure, more confidence, greater freedom in play | | x | x | |
| | Ein größerer Druck das nächste sideout zu machen, da man die Führung nicht verspielen will | Greater pressure to win the next side-out to avoid losing the lead | x | | | |
| | Weniger Druck, befreiteres Aufspielen, selbstbewusster im Angriff, einfacheres abfinden mit Fehlern | Less pressure, freer play, more confident attacking, easier acceptance of errors | | x | | |
| | kann. | You shouldn't ease up but always aim to deliver your best possible performance, even when it's hard | x | | | |
| | Weniger Druck während des Spiels. Ich kann befreiter spielen. | Less pressure during the game. I can play more freely | | x | | |
| | der nächste punkt zählt | The next point matters | x | | | |
| | Das wir gerade besser sind als das andere Team. Wir unsere Stärken weiter ausspielen und die Schwächen weiterhin minimieren wollen und auf Änderungen des Spiels der Gegnerinnen reagieren wollen (wachsam sind). | That we are currently better than the other team. We want to keep using our strengths, minimize weaknesses, and respond to changes in the opponent's game (stay alert) | x | | x | |
| | Konzentration hoch halten und Konsequent bleiben Punkt für Punkt zu Ende spielen | Keep focus high and stay consistent – play point by point until the end | x | | | |
| | Bedeutet für mich nicht viel, da ich mich im Spiel wenig auf den Spielstand konzentriere. Eine hohe Führung für das Team kann zum Spannungsabfall führen und sich auf den nächsten Satz auswirken, es gilt sich daran zu erinnern, weiterhin vollen Fokus zu behalten | Doesn't mean much to me since I don't focus much on the score during the game. A big lead can cause loss of tension and impact the next set, so keep focus | x | | | |
| | 3 Punkte Führung bzw. guter Zugriff/ Read auf das gegnerische Team | 3-point lead or good access/read on the opponent | | | x | |
| | Hoher Fokus auf das eigene Sideout | High focus on our own side-out | x | | | |
| | Wir können uns auch mal einen Fehler erlauben, müssen aber trotzdem sauber zu Ende spielen | We can afford the occasional mistake, but we still need to finish cleanly | | x | | |
| | Große Konzentration auf den eigenen Side out und dabei insbesondere auf die Annahme. Sich eine Taktik zurechtlegen, welche Bälle wie verteidigt werden sollen, weil der Gegner vermeintlich im Stress eher seine Lieblingsbälle spielt. | Strong focus on our own side-out, especially on reception. Prepare a strategy for what balls to defend since the opponent may go for their favorite shots under stress | x | | | |
| | Sicherheit/Bestätigung der besseren Leistung | Security/validation of superior performance | | | x | |
| | dass man gut spielt, jedoch nicht nachlassen sollte und weiterhin so spielen sollte | That you're playing well, but still shouldn't ease up and should continue playing that way | x | | x | |
| | Gegner keine Chance zu geben in sein Spiel reinzukommen. Und somit vielleicht auch eine Unsicherheit in meinem Team auszulösen. | As a team, a big lead means still maintaining intensity and activity on court so the opponent doesn't get back into the game | x | | | |
| | weniger Druck, mehr Lockerheit in allen Aktionen | Less pressure, more ease in all actions | | x | | |
| | sprechen | I consider 3 or more points a big lead. Around 70% win probability feels high | | | | x |
| | Nichts | Nothing | | | | x |
| | Energie oben halten, Spielniveau halten | Keep energy up, maintain playing level | x | | | |
| | mehr Punkte als die Gegner, wärend des Spiels vergangene Spiele gegen Gegner gewonnen oder mehr Erfolge als gegnerische Team Technisch/Taktisch/Athletisch oder Erfahrungsmäßig weiter als Gegner (trägt zu Gewinnwahrscheinlichkeit bei) Zustand Gegner(verletzt/ kaputt von vorhergegangenen Spielen) | More points than the opponent, past wins, more experience, or opponent is exhausted/injured | | | x | |
| | Mehr als 4 Punkte Führung | More than a 4-point lead | | | | x |
| | Befreiteres Aufspielen, mehr Risikomanagement | Freer play, more risk management | | | | x |
| | Hohe Führung, gutes Gefühl am Tag | Big lead, good feeling on the day | | x | x | |
| | Nichts. Genauso weiter spielen wie bisher. | Nothing. Just keep playing as before | | | | x |
| | Eigene Fehler können schneller abgehakt werden. | Own mistakes can be brushed off more easily | x | x | | |
| | Es bedeutet für mich Fokus und Ruhe für den nächsten Punkt und vor allem Selbstvertrauen in das eigene Team. | To me, it means focus and calm for the next point and especially trust in our own team | | | x | |
| | Weiterhin konsquent spielen. Das eigene Spiel nicht vom Spielstand abhängig werden zu lassen. | Stay consistent. Don't let the score affect your own game | x | | | |
| | Ab ca. 70 Prozent. | From about 70 percent | | | | x |
| | Wenn wir im ersten oder zweiten um 5 Punkte führen. | When we lead by 5 points in the first or second set | | | | x |
| | Das man die richtige Taktik hat aber auch das man das Spiel weiter durchziehen muss um den Druck auf den Gegner hochzuhalten | Having the right tactics but also continuing to push to keep pressure on the opponent | x | | | |
| | Gute gewinnaussichten aber auch druck | Good winning prospects but also pressure | | | | x |
| Coaches | - etwas mehr Sicherheit in den eigenen Aktionen | Slightly more confidence in one's actions | | | | x |
| | Völlig abhängig von verschiedenen Faktoren wenn es für mich eine Rolle spielen würde! Keine Bedeutung. Punkt für Punkt zu spielen egal ob Führung oder Gewinnwahrscheinlichkeit. Wenn ich mich im Spiele mit Führung und Wahrscheinlichkeiten beschäftige liegt der Fokus falsch. | Totally depends on various factors if it mattered to me. No meaning. Play point by point regardless of lead or win probability | x | | | |
| | Nicht viel, ich versuche immer den Fokus beim eigenen Können bzw. der eigenen Leistung zu haben. Ich halte nicht viel davon, wenn man Gegner vorher in die eine oder andere Richtung einordnet. | Not much. I always try to focus on my own ability and performance. I don't think much of judging opponents in advance | x | | | |
| | Fokus auf Routinen - es bedeutet nichts | Focus on routines – it means nothing | x | | | |
| | Sicherheit im Sideout --> Kontrolle des Spiels | Security in side-out → control of the game | | | | |
| | beschäftigt = ein positives Gefühl / der eigene Fokus kann mehr auf den Gegner ausgerichtet werden / tendenziell Vorfreude, Euphorie | Tactic and skills executed successfully, opponent is busy dealing with it → positive feeling / more focus on opponent / anticipation and excitement | x | | | |
| | Vertrauen und Spielsicherheit | Trust and game stability | | | | x |
| | Weiter Druck ausüben - nicht nachlassen wegen Führung | Keep applying pressure – don't ease up because of a lead | | | | x |
| | extra ruhe | Extra calm | | x | | |

*Table S6: Comprehensive list and detailed assignment of all answers and themes to question 3*

| **Question 3** | **What strategies are used to compensate for a deficit of 3 or more points?** | | **Serve Pressure** | **Tactical Adjustments** | **Communication and Composure** |
|---|---|---|---|---|---|
| | **Original (GERMAN)** | **translated to English** | | | |
| | Gedankliche Trennung von K1 und K2 intensivieren. Erhöhter Aufschlagdruck um Breakchancen herbei zu führen | Mentally separate K1 and K2 more strongly, increase serve pressure to create break chances | x | | |
| | Lösungsorientiertes Spielen, auf Basics fokussieren | Play solution-oriented, focus on the basics | | x | |
| | Deutlich höherer Aufschlagdruck | Significantly higher serve pressure | x | | |
| | etwas ändern , den Gegner aus dem Rhythmus bringen, taktische Veränderung im Aufschlag zum Beispiel | Change something to disrupt the opponent's rhythm, such as tactical variation in the serve | x | x | |
| | Auszeit nehmen, Zeit verschaffen zwischen den Ballwechseln (Sand schieben, Brille Putzen, eventuell gelbe Karte) | Take a timeout, buy time between rallies (rake sand, clean glasses, possibly accept a yellow card) | | x | |
| | kühlen Kopf bewahren - side-out halten und mehr Taktik im Break absprechen | Keep a cool head – maintain side-out and discuss more tactics during break situations | | x | x |
| | Deutlich mehr Kommunikation im Team, über Tipps für Sideout oder Block/Defensestrategieänderungen, Auszeiten, Passvarianten, unter Drucksituation trotzdem klar bleiben | Clearly more communication within the team, discuss tips for side-out or changes in block/defense strategy, timeouts, passing variants, and staying composed under pressure | | | x |
| | Reflexion des bisherigen Spielverlaufs (wie ist der Rückstand entstanden) und entsprechende Anpassung daran. Eventuell ändern der Taktik. (Anderen Spieler anservieren, Block/ Defence ändern, andere Spots im Aufschlag oder andere Aufschlagart serviere, Rhythmus verändern) | Reflect on how the game has gone so far (why are we trailing) and adjust accordingly. Possibly change tactics (serve different player, adjust block/defense, use different serve zones or types, alter rhythm) | x | x | |
| | Änderung im K2, um Bild für Gegner zu ändern und einen Fehler bzw. eine Break Chance zu provozieren | Change in K2 to alter the image the opponent sees and provoke an error or a break chance | | x | |
| | Mehr Risiko, anderes Muster | More risk, different patterns | | x | |
| | Mehr Risiko im Aufschlag und ggf taktik ändern | More risk on serve and possibly change tactics | x | x | |
| | Hoher Fokus auf den eigenen Aufschlag um das Break zu erleichtern. Mit ruhigere Art und Weise die Ballwechsel spielen und nicht hektisch werden, durch empfunden Druck/Stress. | High focus on your own serve to facilitate a break. Play rallies more calmly and avoid getting hectic due to perceived pressure or stress | x | | |
| | die Probleme die in dem Spiel auftreten zu bemerken und zu verbessern bzw lösungen dafür zu suchen | Notice problems that arise during the game and actively look for solutions | | | x |
| Athletes | Wenn noch keine Auszeit genommen wurde, wird diese gezogen. Und sonst einen Positionswechsel von uns damit der Gegner ein anderes Bild bekommt und nicht seine Standardtaktik durchführen kann. Und dann mit Aufschlagrisiko versuchen die Gegner aus ihrer Komfortzone rauszubringen. | If no timeout has been taken yet, take it now. Possibly change player positions so the opponent gets a different picture and can't follow their standard tactics... | x | | |
| | Taktiken gegen die Taktiken vom Gegner | Tactics against the opponent's tactics | | x | |
| | Aufschlagdruck erhöhen, mehr Risiko eingehen | Increase serve pressure, take more risk | x | | |
| | eventuelle Spielstrategie ändern, Seiten wechseln, Auszeit | Change game strategy if needed, switch sides, take a timeout | | x | |
| | meist andere Abwehrstrategie, die wir im Vorfeld beschsprochen haben, sofern wir merken, dass unsere Abwehrstrategie nicht funktionieren Auf basics konzentrieren | Usually switch to a different defensive strategy if the current one isn't working. Focus on the basics | | x | |
| | Durchatmen, klaren Plan besprechen | Take a breath, discuss a clear plan | | | x |
| | Veränderung, Variation, Gegner was anderes zeigen, um neue Lösungen zu erzwingen | Introduce changes or variation, show something new to the opponent to force new solutions | | x | |
| | Taktikveränderung, mehr Risiko im Aufschlag, Plan B | Tactical change, more serve risk, switch to plan B | x | x | |
| | Evtl. Aufschlagtaktik und Block/Defense verändern. | Possibly change serve tactic and block/defense | x | x | |
| | Vor Augen führen welche Schwachstellen der Gegner hat und dagegen agieren. | Be aware of the opponent's weaknesses and act against them | | x | |
| | Ähnlich wie wenn man vorne steht, versucht man verschiedene Taktiken/Arten von K2, um eine Schwäche des Gegners zu finden, bzw. Sie aus dem Gleichgewicht zu bringen. | Similar to being ahead—try different K2 strategies to find a weakness and throw the opponent off balance | | x | |
| | Variation in die eigene Spielstrategie. Evtl. versuchen zu analysieren wie die vorgenommene Strategie auf den Gegner wirkt und ob nicht vielleicht eine Veränderung der Taktik notwendig ist um den Rückstand wieder aufzuholen. | Vary your own game strategy. Try to analyze how your current strategy affects the opponent and whether a change is needed to recover from the deficit | | x | |
| | Eine angemessene blockfeldabwehr; auf die andere spielerin spielen | Use an appropriate block-defense system; target the other player | | x | |
| | Auch mal einen Aufschlag auf die andere Person machen damit die Person auf die die meisten Bälle kommen in der Annahme nicht zu sicher wird und der andere auch mal überrascht wird | Sometimes serve to the other person so the usual receiver isn't too comfortable and the other one gets surprised | x | | |
| | - mehr Risiko im Aufschlag | More risk on the serve | x | | |
| | hoher risiko erlaubed | High risk is allowed | x | | |
| | Mehr Risiko im Aufschlag | More risk on the serve | x | | |
| | - Aufschlagdruck erhöhen - Taktik anpassen, heißt bspw. anderen Spieler anservieren - Block/Abwehr Taktik/Strategie anpassen | Increase serve pressure – adapt tactics, e.g., serve different player – adjust block/defense strategy | x | | |
| | Abhängig vom Verlauf des Spiels: Stategiewechsel bzw. Anpassung | Depending on the course of the game: strategic change or adjustment | | x | |
| Coaches | Spielverlangsamung, d.h. zwischen den Ballwechseln wird ggf. der Rhythmus verlängert auch auf die Gefahr einer gelben Karte für Spielverzögerung, um Gegner aus dem erfolgreichen Rhythmus zu bringen und selbst Kapazitäten (physisch, psychisch) aufzubauen Analyse, in welchem Bereich Gegner stärker ist, um eigenes Spielsystem entsprechend zu verändern und zu stabilisieren (bspw.: Gegner stark im Aufschlag = Annahmeposition, Annahmeanspruch verändern / Gegner stark im Block = Zuspiele ggf. netzentfernter etc.) | Slow down the game between rallies even at risk of yellow card for delay, to break opponent rhythm and restore your own physical and mental capacity. Analyze where the opponent is stronger and adapt your own system (e.g., change reception or setting based on block strength) | | x | |
| | Hängt natürlich vom konkreten Punktestand ab. Daher nicht eindeutige zu beantworten - Erhöhung Aufschlagdruck - Anspielen anderer Person - Veränderung Block-Feldabwehr | Depends on the exact score. Can't be answered definitively – increase serve pressure – serve different player – change block-defense setup | x | x | |

*Table S7: Comprehensive list and detailed assignment of all answers and themes to question 4*

| Question 4 | What does a high deficit or a low probability of winning mean for you or your team? | | Tactical Adjustments | Mental Focus | Others |
|---|---|---|---|---|---|
| | **Original (GERMAN)** | **translated to English** | | | |
| Athletes | Den Kopf nicht in den Sand stecken. Versuchen das Momentum auf seine Seite zu ziehen und die Emotionen bewusster zu steuern. | Don't bury your head in the sand. Try to shift the momentum to your side and consciously manage your emotions | | x | |
| | versuchen sich nicht vom Punktestand beeinflussen zu lassen, Handlungsorientiert spielen | Try not to let the score affect you—stay action-oriented | | x | |
| | Manchmal frustriert. Wenn man eine Taktik hat, diese zu ändern | Sometimes it's frustrating. If a tactic isn't working, change it | | | x |
| | entspanntes Aufspielen, weniger Druck im einzelnen Ball, eh egal -Einstellung | Play with a relaxed mindset, reduce pressure on individual plays—go in with an "it's okay" attitude | | x | |
| | Trozdem immer alles geben, das Spiel ist noch nicht verloren, alles ist möglich. | Still give it your all, the game isn't lost yet—anything is possible | | x | |
| | Mehr Druck im Spiel. Das wir dass, was wir bisher gemacht haben, besser machen müssen und in bestimmten Elementen noch mehr Fokus setzen müssen. Wie z.B. Aufschlagdruck. | Increase pressure in the game. We need to do what we've been doing even better, and focus more on specific elements, like service pressure | x | | |
| | der nächste punkt zählt - eventuell aufschlagrisiko anpassen. | The next point counts—possibly adjust serving risk | x | | |
| | Darauf reagieren, die Schwächen zu minimieren. Von Punkt zu Punkt schauen. Spielrhythmus unterbrechen und nach mit dem nächsten Ball/ Spielaktion beschäftigen. | Respond by minimizing weaknesses. Go point by point. Disrupt the opponent's rhythm and focus fully on the next rally or play | | x | |
| | Punkt für Punkt spielen, wieder ins Spiel/Flow reinfinden und im Hier und Jetzt bleiben | Play point by point, find your flow again, and stay in the here and now | | x | |
| | Motivation wieder ins Spiel zu kommen, je nach Punktestand Vorbereitung für den nächsten Satz -> Fehlermanagement | Find motivation to get back into the game. Depending on the score, start preparing mentally for the next set—error management | | x | |
| | 3 Punkte Rückstand und/ oder kein Zugriff/ Read auf das gegnerische Team | Being 3 points behind and/or having no read on the opposing team | | | x |
| | Nerven behalten und mutig spielen | Keep your nerve and play boldly | | x | |
| | Mehr Risiko gehen, um zum Erfolg zu kommen. Der Gegner hat den Druck den Satz sauber zu Ende zu bringen | Take more risks to succeed. The opponent is under pressure to close out the set cleanly | x | | |
| | Alles reinwerfen | Throw everything at it | x | x | |
| | Eine besondere Herausforderung-das Leistungsniveau darf nicht weiter abfallen, sondern muss zusätzlich gesteigert werden um den Gegner unter Druck zu setzen | A special challenge—the performance level must not drop any further. In fact, it needs to increase to put the opponent under pressure | | | x |
| | jetzigen Taktiken ändern, konsequenter spielen | Change the current tactics, play more consistently | x | | |
| | Wenn es im ersten Satz ist den Satz abhaken und sich bestmöglich reinspielen für den nächsten Satz. Und nochmal versuchen den Gegner ein wenig unter Druck zu setzten am ende des Satzes um ihm nicht das perfekte Gefühl für den nächsten Satz zu geben. | If it's the first set, put it behind you and focus on getting into rhythm for the next one. Try to put some pressure on the opponent at the end of the set so they don't carry perfect momentum into the next | x | | |
| | Mehr Druck, Verkrampfung, wenig Lockerheit, weniger Beinarbeit, unregelmäßige Streckung beim Zuspiel, Hektik | More pressure, tension, lack of relaxation, reduced footwork, irregular arm extension during setting, hectic play | x | | |
| | 3 Punkte und mehr sind ein hoher Rückstand. Niedrige Siegquote wenn sie bei 55% liegt | A 3-point or greater deficit is considered a significant gap. Win probability drops when it's around 55% | | | x |
| | Stress | Stress | | | x |
| | Spielstrategie funktioniert nicht, Fokus fehlt, fehlende Energie/Stimmung auf dem Feld | Game strategy isn't working, lack of focus, low energy or poor team vibe on the court | | x | |
| | ende des Satzes mehr als 3 Punkte Rückstand bei Aufschlag keinen Zugriff auf Gener(Gegner mit Aufschlag unter Druck sezten/ Abwehrstrategie funktioniert beides nicht) im Vorfeld ; Gegner deutlich erfahrener und bessere Erfolge in der vorhergegangenen Saison | End of the set: more than 3 points behind while serving, no access to opponent's patterns (serve pressure and defensive strategy both not effective); opponent is clearly more experienced and had better results in previous season | x | | |
| | 5 Punkte zurück liegen | Trailing by 5 points | | | |
| | Kreativität, Analyse, neue Wege | Creativity, analysis, and exploring new solutions | x | | |
| | Spiel ausnutzen als Training für neue Herausforderungen, mehr Risiko | Use the match as training for future challenges—take more risks | x | | |
| | Aufschlagdruck erhöhen. Side-Out stabilisieren (andere Routen laufen) | Increase serve pressure. Stabilize side-out by running different attack routes | x | | |
| | Eigenen Sachen nicht gut gemacht , zu wenig Druck auf den Gegner gemacht | Didn't execute our own plays well, applied too little pressure on the opponent | x | | |
| | Wenn das eigene Spiel zu verkrampft oder hektisch wird. Selbstzweifel verursachen, dass man sich zu sehr mit den eigenen Fehlern und mit den Stärken des Gegners beschäftigt. | When your own game becomes too tense or rushed. Self-doubt leads to over-focusing on your own mistakes and the opponent's strengths | | x | |
| | Dass trotzdem alles reingelegt werden muss, um den Satz oder das Spiel noch zu drehen. Den Fokus weniger auf den Punktestand zu setzen sondern eher handlungsorientiert an die nächsten Aktionen ranzugehen. Punkt für Punkt zu spielen. | Still give everything to try and turn the set or the match around. Shift focus away from the score and instead approach each next action in an action-oriented way. Play point by point | | x | |
| | Ca. 30 Prozent. | Approximately 30 percent | | | x |
| | Wenn wir 4 Punkte zurückliegen. | When trailing by 4 points | | | x |
| | Das man eventuell Seine Taktik anpassen muss aber man auch weiterhin befreit und ohne Druck spielen muss | You may need to adjust your tactics but still keep playing freely and without pressure | x | x | |
| | Positive Herangehensweise, an das eigene Können und den Erfolg glauben. Als Team unterstützen und versuchen in eine Flow zu [illegible] | Maintain a positive mindset—believe in your own ability and in success. Support each other as a team and try to get into a flow | | x | |
| Coaches | weniger zeit fur punkte zu nehmen am abwehr | Less time to score points through defense | x | | |
| | Gleiche Antwort wie oben. Im Spiel wäre es der falsche Fokus. Jeder Punkt wird mit allem was ich kann und bereit bin zu geben gespielt. Hohe Rückstände können meist sehr einfache Gründe sein: zu schlecht am Tag x nicht in Form schlechte Umsetzung einer Taktik schlechte Taktik keine Anpassung an äußere Bedingungen schlechte Regulierung .................. und viele Gründe mehr | Same answer as before. In a game, that would be the wrong focus. Every point is played with everything I have and I'm ready to give it my all. Large deficits often have simple explanations: having an off day, not in shape, poor execution of a tactic, bad tactic, lack of adaptation to external conditions, poor self-regulation... and many more reasons | | x | |
| | Grundsätzlich geht es um primär um die eigene Leistung. Ich beschäftige mich nicht dominant mit dem Gegner. Ein hoher Rückstand heißt häufig, dass ich meine Leistung (Sideout) nicht dementsprechend erbringe. | Ultimately, it's primarily about my own performance. I don't focus mainly on the opponent. A significant deficit usually means I'm not performing my own game (side-out) well enough | | | x |
| | Fokus auf Routinen - es bedeutet nichts | Focus on routines—it doesn't mean anything in itself | | x | |
| | Wir müssen etes besser machen oder etwas verändern | We have to do things better or change something | x | | |
| | - Unsicherheit in den eigenen Aktionen | Uncertainty in one's own actions | | x | |
| | Taktik + eigene Fähigkeiten werden nicht erfolgreich abgerufen = Taktikänderung, Technikfokussierung = neutrales Gefühl / realistische Einschätzung der Lage / der eigene Fokus ist überwiegend auf sich ausgerichtet | Tactic + personal skills are not executed successfully = change of tactic, focus on technique = neutral mindset / realistic assessment of the situation / focus is mostly self-directed | x | x | |
| | Lerneffek | Learning effect | | | x |
| | - Risiko erhöhen - Strategie im eigenen Side Out anpassen - von bisheriger Taktik in Block-Feldabwehr evtl abweichen ( wenn Problematik im Break) | Increase risk – adapt strategy in side-out – possibly deviate from previous block-defense tactic (if there are issues during break points) | x | | |
| | Risiko Erhöhung Locker drauf los spielen | Increase risk – play freely and with ease | x | x | |

# 4. Tables and Figures of regression analyses

## a. Figures of all single linear regression models

On the following page, Figure S7 shows the linear regression models of the dependent variable set-winning probability estimation discrepancy (SWP-ED) with the independent variables of our five assessed decision-making tendencies. The models were performed split by our three participant groups and five score categories. A summarizing of the calculated correlations with p-values is given in the manuscript.

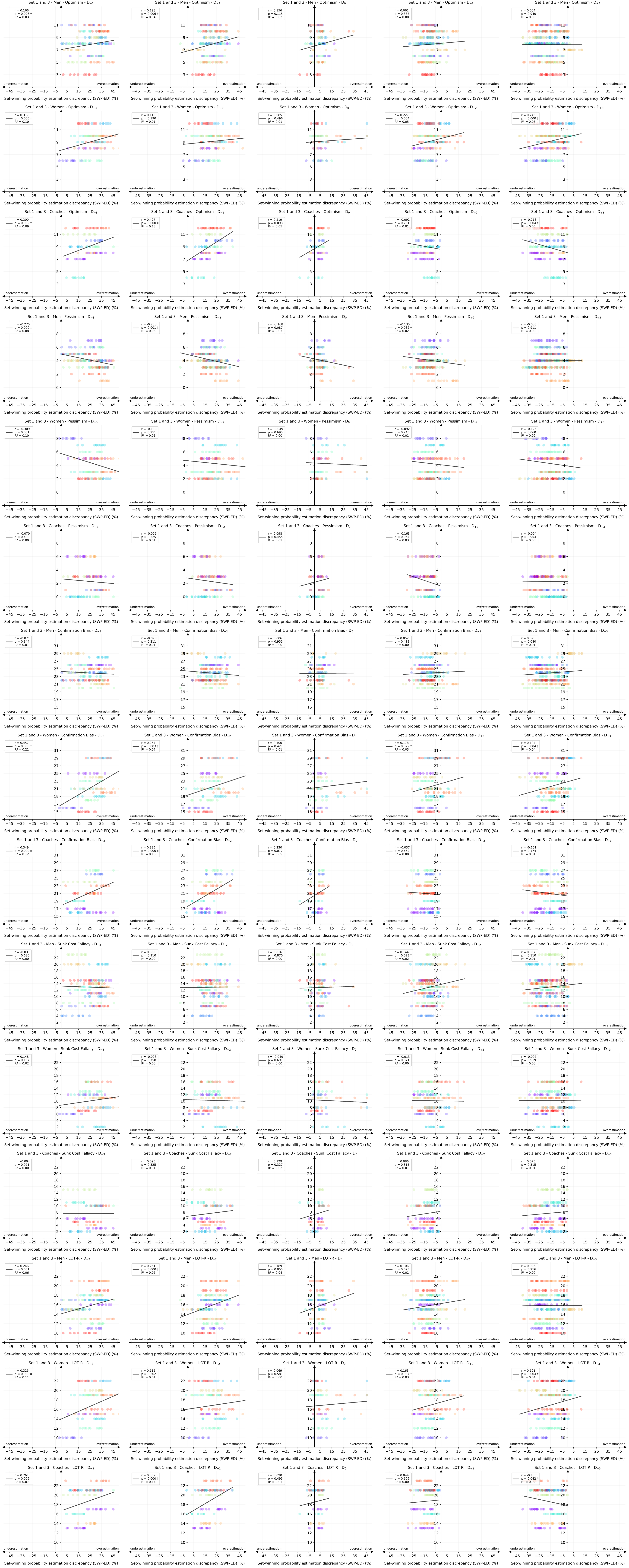

Set 1 and 3 - Men - Optimism - D-3
r = 0.166
p = 0.026 *
R² = 0.03
Set 1 and 3 - Men - Optimism - D-2
r = 0.198
p = 0.006 †
R² = 0.04
Set 1 and 3 - Men - Optimism - D0
r = 0.156
p = 0.115
R² = 0.02
Set 1 and 3 - Men - Optimism - D+2
r = 0.061
p = 0.337
R² = 0.00
Set 1 and 3 - Men - Optimism - D+3
r = 0.004
p = 0.940
R² = 0.00
Set 1 and 3 - Women - Optimism - D-3
r = 0.317
p = 0.000 ‡
R² = 0.10
Set 1 and 3 - Women - Optimism - D-2
r = 0.118
p = 0.190
R² = 0.01
Set 1 and 3 - Women - Optimism - D0
r = 0.085
p = 0.496
R² = 0.01
Set 1 and 3 - Women - Optimism - D+2
r = 0.227
p = 0.004 †
R² = 0.05
Set 1 and 3 - Women - Optimism - D+3
r = 0.245
p = 0.000 ‡
R² = 0.06
Set 1 and 3 - Coaches - Optimism - D-3
r = 0.300
p = 0.002 †
R² = 0.09
Set 1 and 3 - Coaches - Optimism - D-2
r = 0.427
p = 0.000 ‡
R² = 0.18
Set 1 and 3 - Coaches - Optimism - D0
r = 0.219
p = 0.093
R² = 0.05
Set 1 and 3 - Coaches - Optimism - D+2
r = -0.092
p = 0.281
R² = 0.01
Set 1 and 3 - Coaches - Optimism - D+3
r = -0.213
p = 0.004 †
R² = 0.05
Set 1 and 3 - Men - Pessimism - D-3
r = -0.275
p = 0.000 ‡
R² = 0.08
Set 1 and 3 - Men - Pessimism - D-2
r = -0.238
p = 0.001 ‡
R² = 0.06
Set 1 and 3 - Men - Pessimism - D0
r = -0.168
p = 0.087
R² = 0.03
Set 1 and 3 - Men - Pessimism - D+2
r = -0.135
p = 0.032 *
R² = 0.02
Set 1 and 3 - Men - Pessimism - D+3
r = -0.006
p = 0.911
R² = 0.00
Set 1 and 3 - Women - Pessimism - D-3
r = -0.309
p = 0.001 ‡
R² = 0.10
Set 1 and 3 - Women - Pessimism - D-2
r = -0.103
p = 0.251
R² = 0.01
Set 1 and 3 - Women - Pessimism - D0
r = -0.049
p = 0.695
R² = 0.00
Set 1 and 3 - Women - Pessimism - D+2
r = -0.092
p = 0.243
R² = 0.01
Set 1 and 3 - Women - Pessimism - D+3
r = -0.126
p = 0.060
R² = 0.02
Set 1 and 3 - Coaches - Pessimism - D-3
r = -0.070
p = 0.490
R² = 0.00
Set 1 and 3 - Coaches - Pessimism - D-2
r = -0.095
p = 0.325
R² = 0.01
Set 1 and 3 - Coaches - Pessimism - D0
r = 0.098
p = 0.455
R² = 0.01
Set 1 and 3 - Coaches - Pessimism - D+2
r = -0.163
p = 0.054
R² = 0.03
Set 1 and 3 - Coaches - Pessimism - D+3
r = -0.004
p = 0.954
R² = 0.00
Set 1 and 3 - Men - Confirmation Bias - D-3
r = -0.071
p = 0.344
R² = 0.01
Set 1 and 3 - Men - Confirmation Bias - D-2
r = -0.090
p = 0.211
R² = 0.01
Set 1 and 3 - Men - Confirmation Bias - D0
r = 0.006
p = 0.955
R² = 0.00
Set 1 and 3 - Men - Confirmation Bias - D+2
r = 0.052
p = 0.412
R² = 0.00
Set 1 and 3 - Men - Confirmation Bias - D+3
r = 0.095
p = 0.080
R² = 0.01
Set 1 and 3 - Women - Confirmation Bias - D-3
r = 0.457
p = 0.000 ‡
R² = 0.21
Set 1 and 3 - Women - Confirmation Bias - D-2
r = 0.267
p = 0.003 †
R² = 0.07
Set 1 and 3 - Women - Confirmation Bias - D0
r = 0.100
p = 0.421
R² = 0.01
Set 1 and 3 - Women - Confirmation Bias - D+2
r = 0.178
p = 0.022 *
R² = 0.03
Set 1 and 3 - Women - Confirmation Bias - D+3
r = 0.194
p = 0.004 †
R² = 0.04
Set 1 and 3 - Coaches - Confirmation Bias - D-3
r = 0.349
p = 0.000 ‡
R² = 0.12
Set 1 and 3 - Coaches - Confirmation Bias - D-2
r = 0.395
p = 0.000 ‡
R² = 0.16
Set 1 and 3 - Coaches - Confirmation Bias - D0
r = 0.230
p = 0.077
R² = 0.05
Set 1 and 3 - Coaches - Confirmation Bias - D+2
r = -0.037
p = 0.662
R² = 0.00
Set 1 and 3 - Coaches - Confirmation Bias - D+3
r = -0.101
p = 0.174
R² = 0.01
Set 1 and 3 - Men - Sunk Cost Fallacy - D-3
r = -0.031
p = 0.680
R² = 0.00
Set 1 and 3 - Men - Sunk Cost Fallacy - D-2
r = 0.008
p = 0.910
R² = 0.00
Set 1 and 3 - Men - Sunk Cost Fallacy - D0
r = 0.016
p = 0.870
R² = 0.00
Set 1 and 3 - Men - Sunk Cost Fallacy - D+2
r = 0.144
p = 0.023 *
R² = 0.02
Set 1 and 3 - Men - Sunk Cost Fallacy - D+3
r = 0.087
p = 0.110
R² = 0.01
Set 1 and 3 - Women - Sunk Cost Fallacy - D-3
r = 0.148
p = 0.107
R² = 0.02
Set 1 and 3 - Women - Sunk Cost Fallacy - D-2
r = -0.028
p = 0.758
R² = 0.00
Set 1 and 3 - Women - Sunk Cost Fallacy - D0
r = -0.049
p = 0.691
R² = 0.00
Set 1 and 3 - Women - Sunk Cost Fallacy - D+2
r = -0.013
p = 0.871
R² = 0.00
Set 1 and 3 - Women - Sunk Cost Fallacy - D+3
r = -0.007
p = 0.919
R² = 0.00
Set 1 and 3 - Coaches - Sunk Cost Fallacy - D-3
r = -0.004
p = 0.971
R² = 0.00
Set 1 and 3 - Coaches - Sunk Cost Fallacy - D-2
r = 0.095
p = 0.325
R² = 0.01
Set 1 and 3 - Coaches - Sunk Cost Fallacy - D0
r = 0.129
p = 0.327
R² = 0.02
Set 1 and 3 - Coaches - Sunk Cost Fallacy - D+2
r = 0.086
p = 0.315
R² = 0.01
Set 1 and 3 - Coaches - Sunk Cost Fallacy - D+3
r = 0.075
p = 0.315
R² = 0.01
Set 1 and 3 - Men - LOT-R - D-3
r = 0.246
p = 0.001 ‡
R² = 0.06
Set 1 and 3 - Men - LOT-R - D-2
r = 0.251
p = 0.000 ‡
R² = 0.06
Set 1 and 3 - Men - LOT-R - D0
r = 0.189
p = 0.055
R² = 0.04
Set 1 and 3 - Men - LOT-R - D+2
r = 0.106
p = 0.093
R² = 0.01
Set 1 and 3 - Men - LOT-R - D+3
r = 0.006
p = 0.916
R² = 0.00
Set 1 and 3 - Women - LOT-R - D-3
r = 0.325
p = 0.000 ‡
R² = 0.11
Set 1 and 3 - Women - LOT-R - D-2
r = 0.115
p = 0.202
R² = 0.01
Set 1 and 3 - Women - LOT-R - D0
r = 0.069
p = 0.581
R² = 0.00
Set 1 and 3 - Women - LOT-R - D+2
r = 0.163
p = 0.037 *
R² = 0.03
Set 1 and 3 - Women - LOT-R - D+3
r = 0.191
p = 0.004 †
R² = 0.04
Set 1 and 3 - Coaches - LOT-R - D-3
r = 0.261
p = 0.009 †
R² = 0.07
Set 1 and 3 - Coaches - LOT-R - D-2
r = 0.369
p = 0.000 ‡
R² = 0.14
Set 1 and 3 - Coaches - LOT-R - D0
r = 0.090
p = 0.495
R² = 0.01
Set 1 and 3 - Coaches - LOT-R - D+2
r = 0.044
p = 0.606
R² = 0.00
Set 1 and 3 - Coaches - LOT-R - D+3
r = -0.150
p = 0.042 *
R² = 0.02
underestimation
overestimation
Set-winning probability estimation discrepancy (SWP-ED) (%)

On the following page, Figure S8 shows the linear regression models of the dependent variable set-winning probability estimation discrepancy (SWP-ED) with the independent variables of our five assessed decision-making tendencies on the full sample.

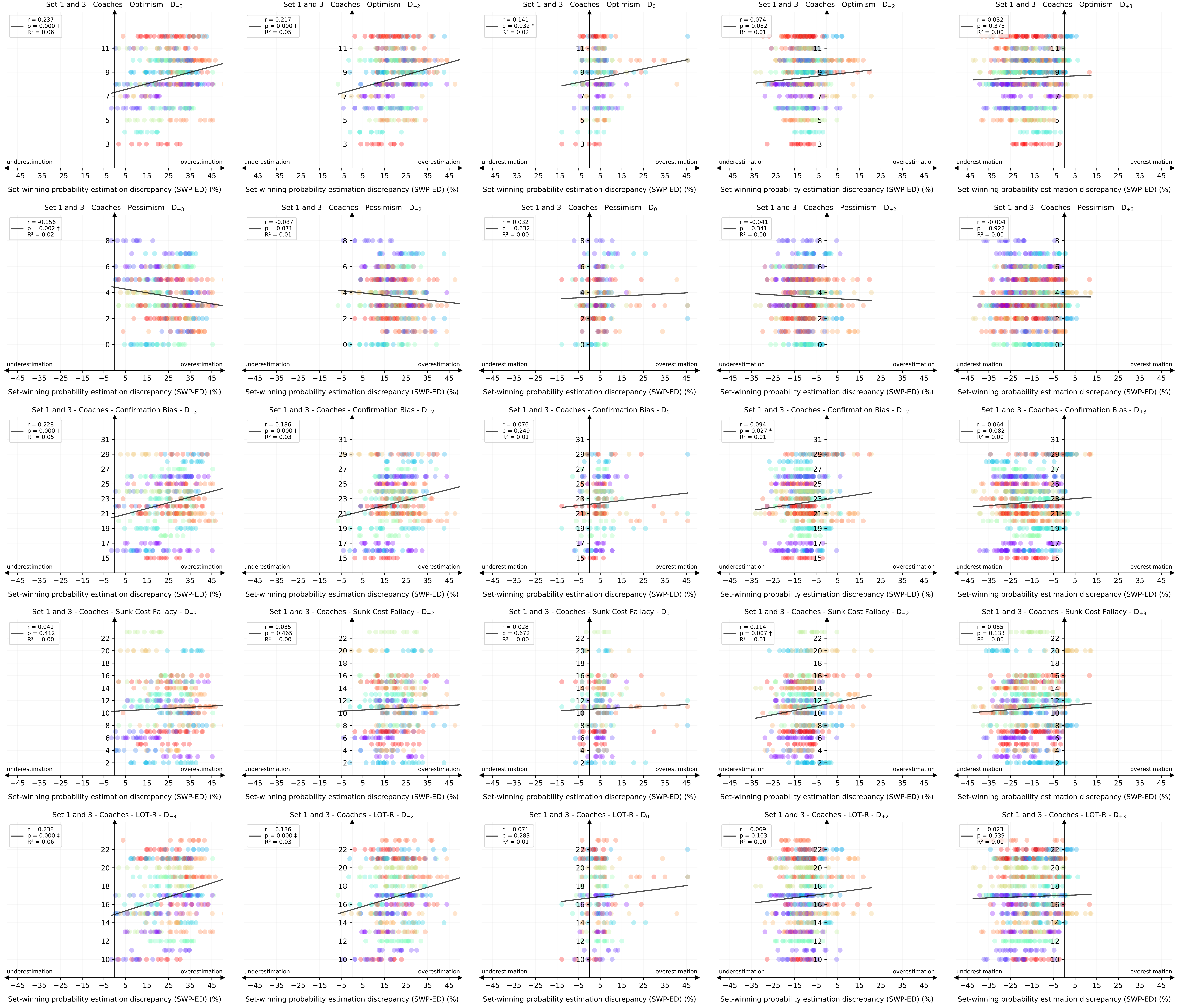
Set 1 and 3 - Coaches - Optimism - D−3
r = 0.237
p = 0.000 ‡
R² = 0.06
Set 1 and 3 - Coaches - Optimism - D−2
r = 0.217
p = 0.000 ‡
R² = 0.05
Set 1 and 3 - Coaches - Optimism - D0
r = 0.141
p = 0.032 *
R² = 0.02
Set 1 and 3 - Coaches - Optimism - D+2
r = 0.074
p = 0.082
R² = 0.01
Set 1 and 3 - Coaches - Optimism - D+3
r = 0.032
p = 0.375
R² = 0.00
Set 1 and 3 - Coaches - Pessimism - D−3
r = -0.156
p = 0.002 †
R² = 0.02
Set 1 and 3 - Coaches - Pessimism - D−2
r = -0.087
p = 0.071
R² = 0.01
Set 1 and 3 - Coaches - Pessimism - D0
r = 0.032
p = 0.632
R² = 0.00
Set 1 and 3 - Coaches - Pessimism - D+2
r = -0.041
p = 0.341
R² = 0.00
Set 1 and 3 - Coaches - Pessimism - D+3
r = -0.004
p = 0.922
R² = 0.00
Set 1 and 3 - Coaches - Confirmation Bias - D−3
r = 0.228
p = 0.000 ‡
R² = 0.05
Set 1 and 3 - Coaches - Confirmation Bias - D−2
r = 0.186
p = 0.000 ‡
R² = 0.03
Set 1 and 3 - Coaches - Confirmation Bias - D0
r = 0.076
p = 0.249
R² = 0.01
Set 1 and 3 - Coaches - Confirmation Bias - D+2
r = 0.094
p = 0.027 *
R² = 0.01
Set 1 and 3 - Coaches - Confirmation Bias - D+3
r = 0.064
p = 0.082
R² = 0.00
Set 1 and 3 - Coaches - Sunk Cost Fallacy - D−3
r = 0.041
p = 0.412
R² = 0.00
Set 1 and 3 - Coaches - Sunk Cost Fallacy - D−2
r = 0.035
p = 0.465
R² = 0.00
Set 1 and 3 - Coaches - Sunk Cost Fallacy - D0
r = 0.028
p = 0.672
R² = 0.00
Set 1 and 3 - Coaches - Sunk Cost Fallacy - D+2
r = 0.114
p = 0.007 †
R² = 0.01
Set 1 and 3 - Coaches - Sunk Cost Fallacy - D+3
r = 0.055
p = 0.133
R² = 0.00
Set 1 and 3 - Coaches - LOT-R - D−3
r = 0.238
p = 0.000 ‡
R² = 0.06
Set 1 and 3 - Coaches - LOT-R - D−2
r = 0.186
p = 0.000 ‡
R² = 0.03
Set 1 and 3 - Coaches - LOT-R - D0
r = 0.071
p = 0.283
R² = 0.01
Set 1 and 3 - Coaches - LOT-R - D+2
r = 0.069
p = 0.103
R² = 0.00
Set 1 and 3 - Coaches - LOT-R - D+3
r = 0.023
p = 0.539
R² = 0.00
underestimation
overestimation
−45 −35 −25 −15 −5 5 15 25 35 45
Set-winning probability estimation discrepancy (SWP-ED) (%)

## b. Complete results of robust linear models

On the following page, Table S8 shows the complete results of all trained robust linear models (RLMs) with the assessed biases (optimism, pessimism, confirmation bias, sunk cost fallacy) as independent variables and the set-winning probability estimation discrepancy (SWP-ED) as dependent variable. A selection of those RLMs with a significant β-value is shown in the manuscript.

*Table S8: Complete results of all trained robust linear models (RLMs) with the assessed biases (optimism, pessimism, confirmation bias, sunk cost fallacy)*

| | | | | const | | | | | | | Optimism | | | | | | | Pessimism | | | | | | | Confirmation | | | | | | | Sunk Cost | | | | | | |
|---|---|---|---|---|---|---|---|---|---|---|---|---|---|---|---|---|---|---|---|---|---|---|---|---|---|---|---|---|---|---|---|---|---|---|---|---|---|---|
| **Model** | **Pseudo-R-squared** | **Scale** | **Observations** | **coef** | **std err** | **t** | **P>\|t\|** | **[0.025** | **0.975]** | **sig** | **coef** | **std err** | **t** | **P>\|t\|** | **[0.025** | **0.975]** | **sig** | **coef** | **std err** | **t** | **P>\|t\|** | **[0.025** | **0.975]** | **sig** | **coef** | **std err** | **t** | **P>\|t\|** | **[0.025** | **0.975]** | **sig** | **coef** | **std err** | **t** | **P>\|t\|** | **[0.025** | **0.975]** | **sig** |
| Male Players - All Scores | 0,01 | 23,62 | 1077 | -3,223 | 7,436 | -0,433 | 0,665 | -17,798 | 11,352 | | 0,380 | 0,406 | 0,937 | 0,349 | -0,416 | 1,176 | | -0,554 | 0,603 | -0,919 | 0,358 | -1,736 | 0,628 | | 0,036 | 0,337 | 0,107 | 0,915 | -0,624 | 0,696 | | 0,166 | 0,198 | 0,835 | 0,404 | -0,223 | 0,554 | |
| Male Players - Trailing (-4 to -3) | 0,09 | 11,01 | 180 | 37,127 | 9,929 | 3,739 | 0,000 | 17,666 | 56,588 | *** | 0,018 | 0,542 | 0,033 | 0,974 | -1,045 | 1,081 | | -2,363 | 0,805 | -2,935 | 0,003 | -3,940 | -0,785 | ** | 0,086 | 0,450 | 0,191 | 0,849 | -0,796 | 0,967 | | -0,298 | 0,265 | -1,125 | 0,261 | -0,817 | 0,221 | |
| Male Players - Trailing (-2 to -1) | 0,08 | 8,62 | 197 | 23,697 | 6,755 | 3,508 | 0,000 | 10,457 | 36,937 | *** | 0,589 | 0,369 | 1,596 | 0,110 | -0,134 | 1,312 | | -0,948 | 0,548 | -1,732 | 0,083 | -2,022 | 0,125 | | -0,260 | 0,306 | -0,851 | 0,395 | -0,860 | 0,339 | | 0,156 | 0,180 | 0,866 | 0,387 | -0,197 | 0,509 | |
| Male Players - Tie | 0,06 | 3,84 | 107 | -1,853 | 5,920 | -0,313 | 0,754 | -13,457 | 9,751 | | 0,203 | 0,323 | 0,628 | 0,530 | -0,431 | 0,837 | | -0,266 | 0,480 | -0,554 | 0,580 | -1,207 | 0,675 | | 0,232 | 0,268 | 0,866 | 0,387 | -0,294 | 0,758 | | 0,060 | 0,158 | 0,377 | 0,706 | -0,250 | 0,369 | |
| Male Players - Leading (+1 bis +2) | 0,03 | 6,03 | 252 | -15,471 | 5,120 | -3,022 | 0,003 | -25,507 | -5,436 | ** | 0,488 | 0,280 | 1,746 | 0,081 | -0,060 | 1,036 | | 0,293 | 0,415 | 0,706 | 0,480 | -0,520 | 1,107 | | -0,192 | 0,232 | -0,827 | 0,409 | -0,646 | 0,263 | | 0,337 | 0,137 | 2,470 | 0,014 | 0,070 | 0,605 | * |
| Male Players - Leading (+3 bis +6) | 0,01 | 11,22 | 341 | -23,444 | 6,998 | -3,350 | 0,001 | -37,159 | -9,729 | ** | 0,220 | 0,382 | 0,575 | 0,565 | -0,529 | 0,969 | | 0,251 | 0,567 | 0,443 | 0,658 | -0,861 | 1,363 | | 0,062 | 0,317 | 0,195 | 0,845 | -0,559 | 0,683 | | 0,165 | 0,187 | 0,881 | 0,378 | -0,201 | 0,530 | |
| Female Players - All Scores | 0,03 | 23,14 | 710 | -37,142 | 15,161 | -2,450 | 0,014 | -66,858 | -7,427 | * | 2,288 | 1,018 | 2,246 | 0,025 | 0,291 | 4,284 | * | 1,298 | 1,013 | 1,281 | 0,200 | -0,687 | 3,284 | | 0,613 | 0,199 | 3,078 | 0,002 | 0,223 | 1,004 | ** | 0,001 | 0,281 | 0,005 | 0,996 | -0,550 | 0,553 | |
| Female Players - Trailing (-4 to -3) | 0,31 | 9,98 | 120 | -11,853 | 17,506 | -0,677 | 0,498 | -46,163 | 22,458 | | 1,668 | 1,175 | 1,419 | 0,156 | -0,636 | 3,972 | | -0,136 | 1,171 | -0,116 | 0,907 | -2,431 | 2,158 | | 1,213 | 0,229 | 5,304 | 0,000 | 0,765 | 1,661 | *** | -0,161 | 0,324 | -0,495 | 0,621 | -0,796 | 0,475 | |
| Female Players - Trailing (-2 to -1) | 0,16 | 10,38 | 129 | 5,462 | 18,423 | 0,296 | 0,767 | -30,646 | 41,571 | | 0,291 | 1,237 | 0,235 | 0,814 | -2,134 | 2,716 | | -0,691 | 1,230 | -0,562 | 0,574 | -3,101 | 1,719 | | 1,068 | 0,244 | 4,383 | 0,000 | 0,590 | 1,545 | *** | -0,680 | 0,343 | -1,984 | 0,047 | -1,351 | -0,008 | * |
| Female Players - Tie | 0,01 | 5,79 | 69 | -4,159 | 17,479 | -0,238 | 0,812 | -38,417 | 30,099 | | 0,373 | 1,174 | 0,318 | 0,751 | -1,928 | 2,674 | | 0,613 | 1,165 | 0,526 | 0,599 | -1,670 | 2,896 | | 0,247 | 0,234 | 1,058 | 0,290 | -0,211 | 0,705 | | -0,123 | 0,326 | -0,377 | 0,707 | -0,762 | 0,517 | |
| Female Players - Leading (+1 bis +2) | 0,12 | 8,15 | 166 | -63,669 | 12,712 | -5,009 | 0,000 | -88,584 | -38,754 | *** | 3,501 | 0,854 | 4,099 | 0,000 | 1,827 | 5,176 | *** | 2,778 | 0,850 | 3,268 | 0,001 | 1,112 | 4,444 | ** | 0,369 | 0,167 | 2,214 | 0,027 | 0,042 | 0,695 | * | 0,274 | 0,236 | 1,163 | 0,245 | -0,188 | 0,736 | |
| Female Players - Leading (+3 bis +6) | 0,14 | 10,26 | 226 | -68,408 | 12,501 | -5,472 | 0,000 | -92,909 | -43,907 | *** | 3,437 | 0,840 | 4,092 | 0,000 | 1,791 | 5,083 | *** | 2,398 | 0,836 | 2,868 | 0,004 | 0,759 | 4,036 | ** | 0,495 | 0,164 | 3,022 | 0,003 | 0,174 | 0,815 | ** | 0,197 | 0,232 | 0,850 | 0,396 | -0,257 | 0,651 | |
| Coaches - All Scores | 0,00 | 21,56 | 600 | -6,895 | 5,412 | -1,274 | 0,203 | -17,502 | 3,711 | | -0,043 | 0,412 | -0,105 | 0,916 | -0,851 | 0,765 | | -0,080 | 0,391 | -0,204 | 0,838 | -0,846 | 0,687 | | 0,299 | 0,348 | 0,860 | 0,390 | -0,382 | 0,980 | | -0,060 | 0,298 | -0,200 | 0,841 | -0,643 | 0,524 | |
| Coaches - Trailing (-4 to -3) | 0,21 | 7,04 | 100 | -8,091 | 5,931 | -1,364 | 0,173 | -19,715 | 3,533 | | 0,561 | 0,452 | 1,241 | 0,214 | -0,325 | 1,447 | | -0,046 | 0,429 | -0,108 | 0,914 | -0,886 | 0,794 | | 1,546 | 0,381 | 4,056 | 0,000 | 0,799 | 2,292 | *** | -0,961 | 0,326 | -2,945 | 0,003 | -1,601 | -0,321 | ** |
| Coaches - Trailing (-2 to -1) | 0,22 | 6,71 | 110 | -7,572 | 5,045 | -1,501 | 0,133 | -17,461 | 2,316 | | 0,931 | 0,384 | 2,423 | 0,015 | 0,178 | 1,685 | * | -0,174 | 0,365 | -0,476 | 0,634 | -0,888 | 0,541 | | 0,967 | 0,324 | 2,982 | 0,003 | 0,331 | 1,602 | ** | -0,515 | 0,278 | -1,853 | 0,064 | -1,059 | 0,030 | |
| Coaches - Tie | 0,11 | 2,97 | 60 | -4,627 | 3,367 | -1,374 | 0,169 | -11,225 | 1,972 | | 0,071 | 0,257 | 0,278 | 0,781 | -0,431 | 0,574 | | 0,362 | 0,243 | 1,488 | 0,137 | -0,115 | 0,839 | | 0,283 | 0,216 | 1,309 | 0,191 | -0,141 | 0,707 | | 0,065 | 0,185 | 0,353 | 0,724 | -0,298 | 0,429 | |
| Coaches - Leading (+1 bis +2) | 0,04 | 5,74 | 140 | -5,355 | 3,696 | -1,449 | 0,147 | -12,598 | 1,888 | | -0,044 | 0,282 | -0,157 | 0,875 | -0,596 | 0,508 | | -0,431 | 0,267 | -1,613 | 0,107 | -0,954 | 0,093 | | -0,364 | 0,237 | -1,535 | 0,125 | -0,830 | 0,101 | | 0,268 | 0,203 | 1,317 | 0,188 | -0,131 | 0,666 | |
| Coaches - Leading (+3 bis +6) | 0,06 | 10,08 | 190 | -8,121 | 5,135 | -1,582 | 0,114 | -18,184 | 1,943 | | -0,858 | 0,391 | -2,192 | 0,028 | -1,624 | -0,091 | * | 0,045 | 0,371 | 0,121 | 0,903 | -0,682 | 0,772 | | -0,247 | 0,330 | -0,749 | 0,454 | -0,894 | 0,399 | | 0,437 | 0,283 | 1,548 | 0,122 | -0,116 | 0,991 | |

*Note: Asterisks (*, p < .05), (**, p < .01), or (***, p < .001) indicate significant Pearson correlations.*

On the following page, Table S9 shows the complete results of all trained robust linear models (RLMs) on the full sample with the assessed biases (optimism, pessimism, confirmation bias, sunk cost fallacy) as independent variables and the set-winning probability estimation discrepancy (SWP-ED) as dependent variable.

*Table S9: Complete results of all trained robust linear models (RLMs) with the assessed biases (optimism, pessimism, confirmation bias, sunk cost fallacy)*

| | | | | const | | | | | | | Optimism | | | | | | | Pessimism | | | | | | | Confirmation | | | | | | | Sunk Cost | | | | | | |
|---|---|---|---|---|---|---|---|---|---|---|---|---|---|---|---|---|---|---|---|---|---|---|---|---|---|---|---|---|---|---|---|---|---|---|---|---|---|---|
| Model | Pseudo-R-squared | Scale | Observations | coef | std err | t | P>\|t\| | [0.025 | 0.975] | sig | coef | std err | t | P>\|t\| | [0.025 | 0.975] | sig | coef | std err | t | P>\|t\| | [0.025 | 0.975] | sig | coef | std err | t | P>\|t\| | [0.025 | 0.975] | sig | coef | std err | t | P>\|t\| | [0.025 | 0.975] | sig |
| full sample - All Scores | 0,01 | 22,96 | 2387 | -11,20 | 3,47 | -3,23 | 0,00 | -18,00 | -4,41 | ** | 0,56 | 0,23 | 2,48 | 0,01 | 0,12 | 1,00 | * | 0,03 | 0,24 | 0,15 | 0,88 | -0,43 | 0,50 | | 0,25 | 0,14 | 1,78 | 0,08 | -0,03 | 0,53 | | 0,11 | 0,11 | 0,95 | 0,34 | -0,11 | 0,33 | |
| full sample - Trailing (-4 to -3) | 0,11 | 10,59 | 400 | 0,05 | 4,41 | 0,01 | 0,99 | -8,60 | 8,71 | | 1,10 | 0,29 | 3,81 | 0,00 | 0,53 | 1,67 | *** | -0,21 | 0,31 | -0,69 | 0,49 | -0,81 | 0,39 | | 0,78 | 0,18 | 4,32 | 0,00 | 0,43 | 1,14 | *** | -0,07 | 0,14 | -0,50 | 0,62 | -0,36 | 0,21 | |
| full sample - Trailing (-2 to -1) | 0,09 | 8,62 | 436 | -0,37 | 3,63 | -0,10 | 0,92 | -7,49 | 6,74 | | 0,84 | 0,24 | 3,57 | 0,00 | 0,38 | 1,31 | *** | -0,03 | 0,25 | -0,11 | 0,92 | -0,52 | 0,46 | | 0,57 | 0,15 | 3,80 | 0,00 | 0,27 | 0,86 | *** | 0,02 | 0,12 | 0,17 | 0,87 | -0,21 | 0,25 | |
| full sample - Tie | 0,03 | 4,28 | 236 | -4,29 | 2,98 | -1,44 | 0,15 | -10,13 | 1,54 | | 0,30 | 0,19 | 1,54 | 0,12 | -0,08 | 0,68 | | 0,41 | 0,21 | 2,02 | 0,04 | 0,01 | 0,82 | * | 0,17 | 0,12 | 1,36 | 0,17 | -0,07 | 0,41 | | 0,10 | 0,10 | 1,00 | 0,32 | -0,09 | 0,29 | |
| full sample - Leading (+1 bis +2) | 0,02 | 6,48 | 558 | -15,36 | 2,65 | -5,81 | 0,00 | -20,55 | -10,18 | *** | 0,30 | 0,17 | 1,73 | 0,08 | -0,04 | 0,64 | | 0,04 | 0,18 | 0,21 | 0,83 | -0,32 | 0,40 | | 0,00 | 0,11 | 0,04 | 0,97 | -0,21 | 0,22 | | 0,17 | 0,09 | 1,94 | 0,05 | 0,00 | 0,34 | |
| full sample - Leading (+3 bis +6) | 0,01 | 11,15 | 757 | -20,80 | 3,22 | -6,46 | 0,00 | -27,10 | -14,49 | *** | 0,18 | 0,21 | 0,88 | 0,38 | -0,23 | 0,60 | | 0,03 | 0,22 | 0,12 | 0,90 | -0,41 | 0,46 | | 0,09 | 0,13 | 0,71 | 0,48 | -0,17 | 0,35 | | 0,07 | 0,11 | 0,68 | 0,50 | -0,13 | 0,28 | |

*Note: Asterisks (*, p < .05), (**, p < .01), or (***, p < .001) indicate significant Pearson correlations.*

# 5. Results for alternative outlier handling

In the following the results of alternative outlier handling are presented. Note, no significant differences could be observed among the three applied outlier handling approaches (customized, winsorizing, and no outlier handling).

## a. Winsorizing approach

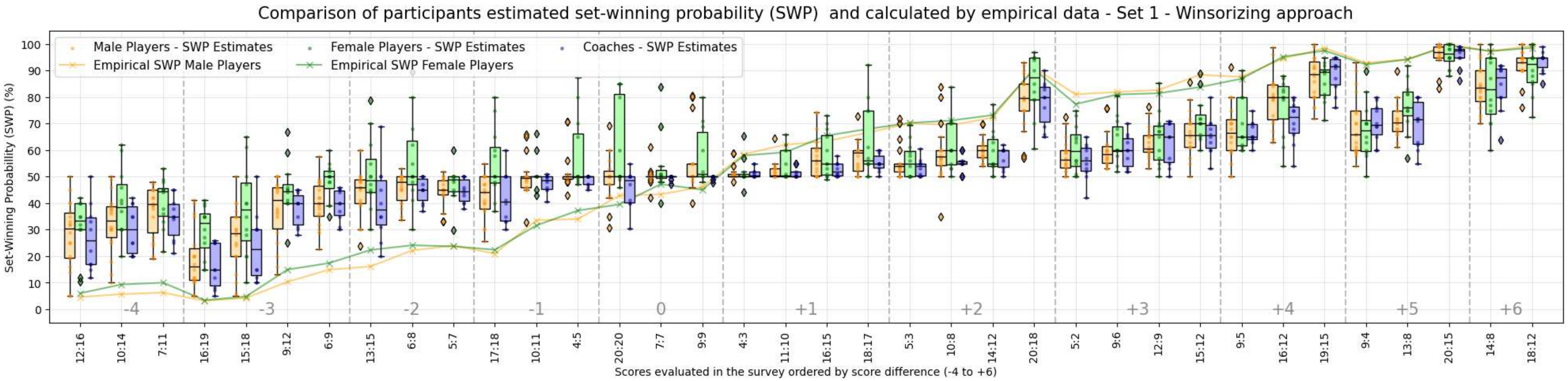


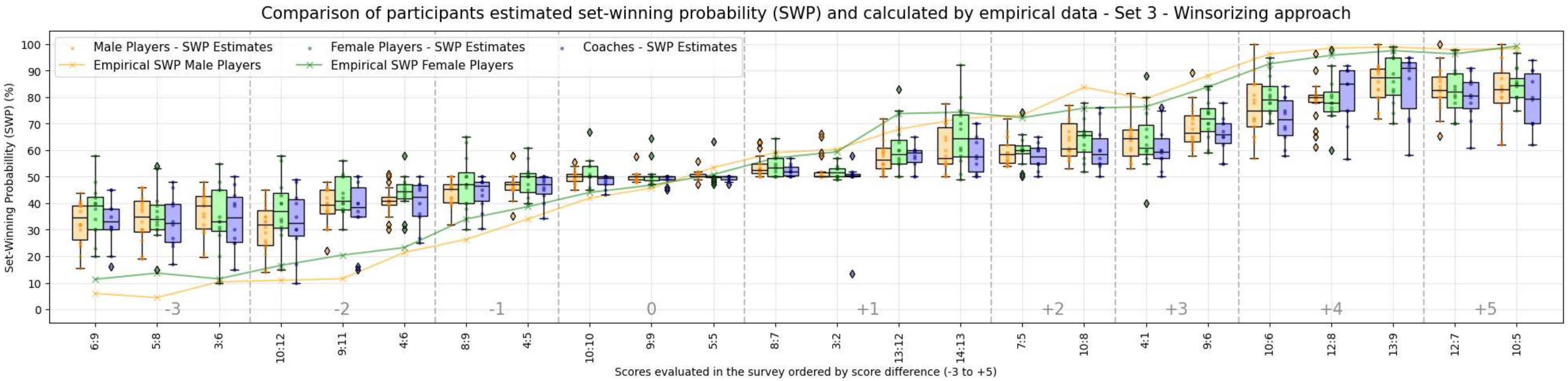


*Figure S9: Participants estimated SWPs compared to empirical calculated SWPs for each evaluated score in the first set (top) and the third set (bottom) of the survey with alternative winsorizing approach as outlier handling. Scores on the y-axis are ordered by the score difference of each score for better readability and do not represent the order in the survey. For coaches, both sexes are displayed together, as there is only one female coach in the sample*

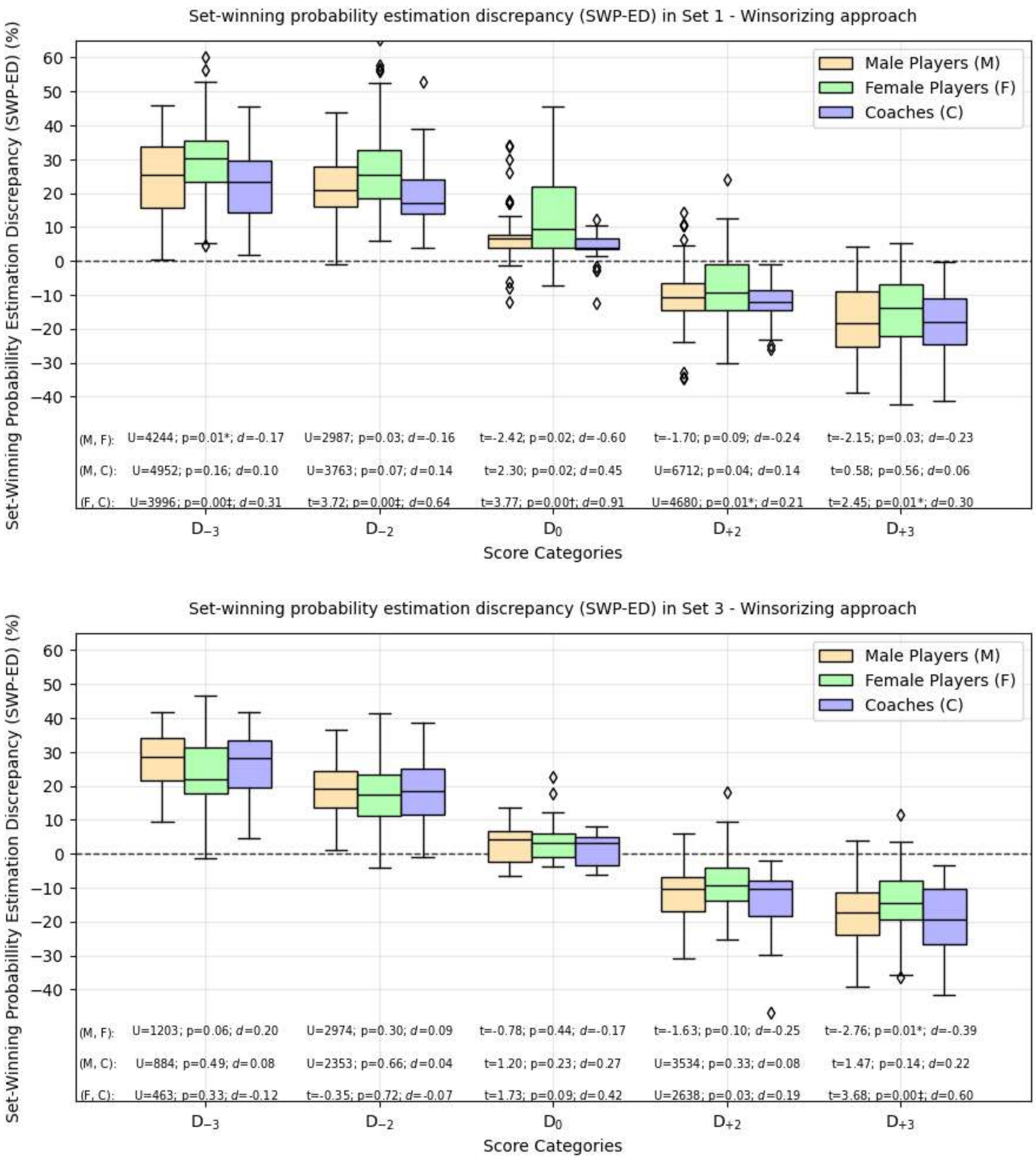


*Figure S10: Set-winning probability estimation discrepancies (SWP-ED) of participants in the first set (top) and third set (bottom) across five score categories with alternative winsorizing approach as outlier handling. The red dotted line indicates perfect estimation without any discrepancy, whereas estimation above indicates over- and below underestimation of the SWP. The SWP-ED is calculated as the difference between a participant's SWP estimate and the empirically calculated SWP for the corresponding score. Independent t-tests or Man-Whitney-U tests were conducted for group comparisons within each score category. To account for multiple comparisons, a Bonferroni correction was applied, adjusting the significance level to α / m, where m is the number of tests conducted per score category. Significant results are marked with asterisk (*, p < .05 / m), dagger (†, p < .01 / m), or double dagger (‡, p < .001 / m).*

*Table S10: Pearson correlation (r) results of Linear Regression models for each participant group (M, F, C), score category, and assessed decision-making tendency as independent variable with alternative winsorizing approach as outlier handling.*

| | Optimism | | | Pessimism | | | Confirmation Bias | | | Sunk Cost Fallacy | | | LOT-R | | |
|---|---|---|---|---|---|---|---|---|---|---|---|---|---|---|---|
| | M | F | C | M | F | C | M | F | C | M | F | C | M | F | C |
| $D_{-3}$ | **.17*** | **.33‡** | **.30†** | **-.29‡** | **-.32‡** | -.07 | -.08 | **.47‡** | **.35‡** | -.03 | .13 | -.00 | **.26‡** | **.34‡** | **.26†** |
| $D_{-2}$ | **.20†** | .17 | **.40‡** | **-.27‡** | -.14 | -.13 | -.07 | **.31‡** | **.42‡** | .04 | -.06 | .11 | **.27‡** | .16 | **.37‡** |
| $D_0$ | .12 | .06 | .22 | -.17 | -.04 | .09 | .11 | .09 | .22 | .10 | -.07 | .12 | .16 | .05 | .09 |
| $D_{+2}$ | .05 | **.23†** | -.08 | -.08 | -.10 | -.11 | .06 | .15 | -.09 | **.13*** | -.02 | .06 | .07 | **.17*** | .01 |
| $D_{+3}$ | -.00 | **.25‡** | **-.22†** | .01 | -.13 | -.01 | .06 | **.20†** | -.07 | .06 | -.00 | .07 | -.01 | **.19†** | **-.15*** |

*Note: In bold, an asterisk (*, p < .05), dagger (†, p < .01), or double dagger (‡, p < .001) indicate significant Pearson correlations. For trailing scenarios ($D_{-3}$ and $D_{-2}$), a negative correlation indicates that higher values of the independent variable led to better estimates, as participants tended to overestimate the SWP. For leading scenarios ($D_{+2}$ and $D_{+3}$), a negative correlation suggests more estimation error or higher underestimation if the independent variable is higher, as participants in our survey predominantly underestimated the SWP.*

On the next page, Figure S11 shows the results of the single linear regression with the winsorizing approach applied.

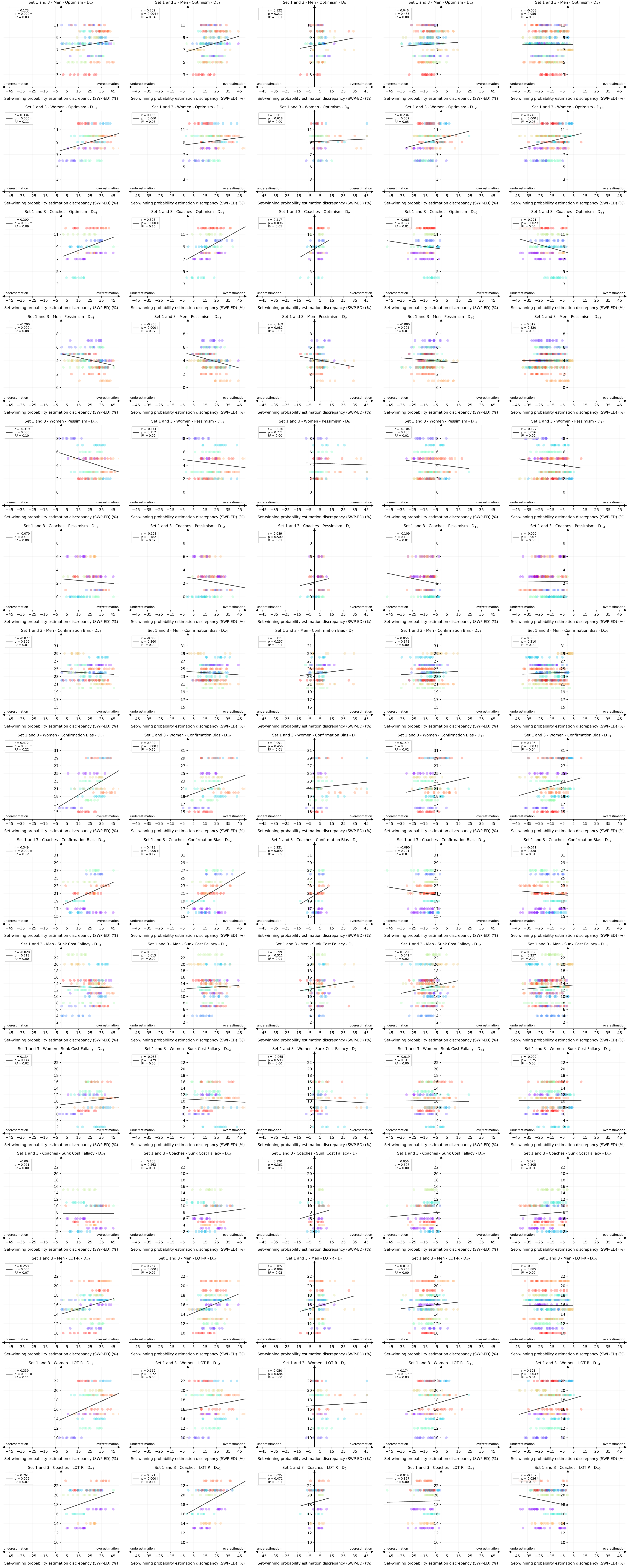
Set 1 and 3 - Men - Optimism - $D_{-3}$
Set 1 and 3 - Men - Optimism - $D_{-2}$
Set 1 and 3 - Men - Optimism - $D_{0}$
Set 1 and 3 - Men - Optimism - $D_{+2}$
Set 1 and 3 - Men - Optimism - $D_{+3}$
Set 1 and 3 - Women - Optimism - $D_{-3}$
Set 1 and 3 - Women - Optimism - $D_{-2}$
Set 1 and 3 - Women - Optimism - $D_{0}$
Set 1 and 3 - Women - Optimism - $D_{+2}$
Set 1 and 3 - Women - Optimism - $D_{+3}$
Set 1 and 3 - Coaches - Optimism - $D_{-3}$
Set 1 and 3 - Coaches - Optimism - $D_{-2}$
Set 1 and 3 - Coaches - Optimism - $D_{0}$
Set 1 and 3 - Coaches - Optimism - $D_{+2}$
Set 1 and 3 - Coaches - Optimism - $D_{+3}$
Set 1 and 3 - Men - Pessimism - $D_{-3}$
Set 1 and 3 - Men - Pessimism - $D_{-2}$
Set 1 and 3 - Men - Pessimism - $D_{0}$
Set 1 and 3 - Men - Pessimism - $D_{+2}$
Set 1 and 3 - Men - Pessimism - $D_{+3}$
Set 1 and 3 - Women - Pessimism - $D_{-3}$
Set 1 and 3 - Women - Pessimism - $D_{-2}$
Set 1 and 3 - Women - Pessimism - $D_{0}$
Set 1 and 3 - Women - Pessimism - $D_{+2}$
Set 1 and 3 - Women - Pessimism - $D_{+3}$
Set 1 and 3 - Coaches - Pessimism - $D_{-3}$
Set 1 and 3 - Coaches - Pessimism - $D_{-2}$
Set 1 and 3 - Coaches - Pessimism - $D_{0}$
Set 1 and 3 - Coaches - Pessimism - $D_{+2}$
Set 1 and 3 - Coaches - Pessimism - $D_{+3}$
Set 1 and 3 - Men - Confirmation Bias - $D_{-3}$
Set 1 and 3 - Men - Confirmation Bias - $D_{-2}$
Set 1 and 3 - Men - Confirmation Bias - $D_{0}$
Set 1 and 3 - Men - Confirmation Bias - $D_{+2}$
Set 1 and 3 - Men - Confirmation Bias - $D_{+3}$
Set 1 and 3 - Women - Confirmation Bias - $D_{-3}$
Set 1 and 3 - Women - Confirmation Bias - $D_{-2}$
Set 1 and 3 - Women - Confirmation Bias - $D_{0}$
Set 1 and 3 - Women - Confirmation Bias - $D_{+2}$
Set 1 and 3 - Women - Confirmation Bias - $D_{+3}$
Set 1 and 3 - Coaches - Confirmation Bias - $D_{-3}$
Set 1 and 3 - Coaches - Confirmation Bias - $D_{-2}$
Set 1 and 3 - Coaches - Confirmation Bias - $D_{0}$
Set 1 and 3 - Coaches - Confirmation Bias - $D_{+2}$
Set 1 and 3 - Coaches - Confirmation Bias - $D_{+3}$
Set 1 and 3 - Men - Sunk Cost Fallacy - $D_{-3}$
Set 1 and 3 - Men - Sunk Cost Fallacy - $D_{-2}$
Set 1 and 3 - Men - Sunk Cost Fallacy - $D_{0}$
Set 1 and 3 - Men - Sunk Cost Fallacy - $D_{+2}$
Set 1 and 3 - Men - Sunk Cost Fallacy - $D_{+3}$
Set 1 and 3 - Women - Sunk Cost Fallacy - $D_{-3}$
Set 1 and 3 - Women - Sunk Cost Fallacy - $D_{-2}$
Set 1 and 3 - Women - Sunk Cost Fallacy - $D_{0}$
Set 1 and 3 - Women - Sunk Cost Fallacy - $D_{+2}$
Set 1 and 3 - Women - Sunk Cost Fallacy - $D_{+3}$
Set 1 and 3 - Coaches - Sunk Cost Fallacy - $D_{-3}$
Set 1 and 3 - Coaches - Sunk Cost Fallacy - $D_{-2}$
Set 1 and 3 - Coaches - Sunk Cost Fallacy - $D_{0}$
Set 1 and 3 - Coaches - Sunk Cost Fallacy - $D_{+2}$
Set 1 and 3 - Coaches - Sunk Cost Fallacy - $D_{+3}$
Set 1 and 3 - Men - LOT-R - $D_{-3}$
Set 1 and 3 - Men - LOT-R - $D_{-2}$
Set 1 and 3 - Men - LOT-R - $D_{0}$
Set 1 and 3 - Men - LOT-R - $D_{+2}$
Set 1 and 3 - Men - LOT-R - $D_{+3}$
Set 1 and 3 - Women - LOT-R - $D_{-3}$
Set 1 and 3 - Women - LOT-R - $D_{-2}$
Set 1 and 3 - Women - LOT-R - $D_{0}$
Set 1 and 3 - Women - LOT-R - $D_{+2}$
Set 1 and 3 - Women - LOT-R - $D_{+3}$
Set 1 and 3 - Coaches - LOT-R - $D_{-3}$
Set 1 and 3 - Coaches - LOT-R - $D_{-2}$
Set 1 and 3 - Coaches - LOT-R - $D_{0}$
Set 1 and 3 - Coaches - LOT-R - $D_{+2}$
Set 1 and 3 - Coaches - LOT-R - $D_{+3}$
underestimation
overestimation
Set-winning probability estimation discrepancy (SWP-ED) (%)

## b. No outlier handling

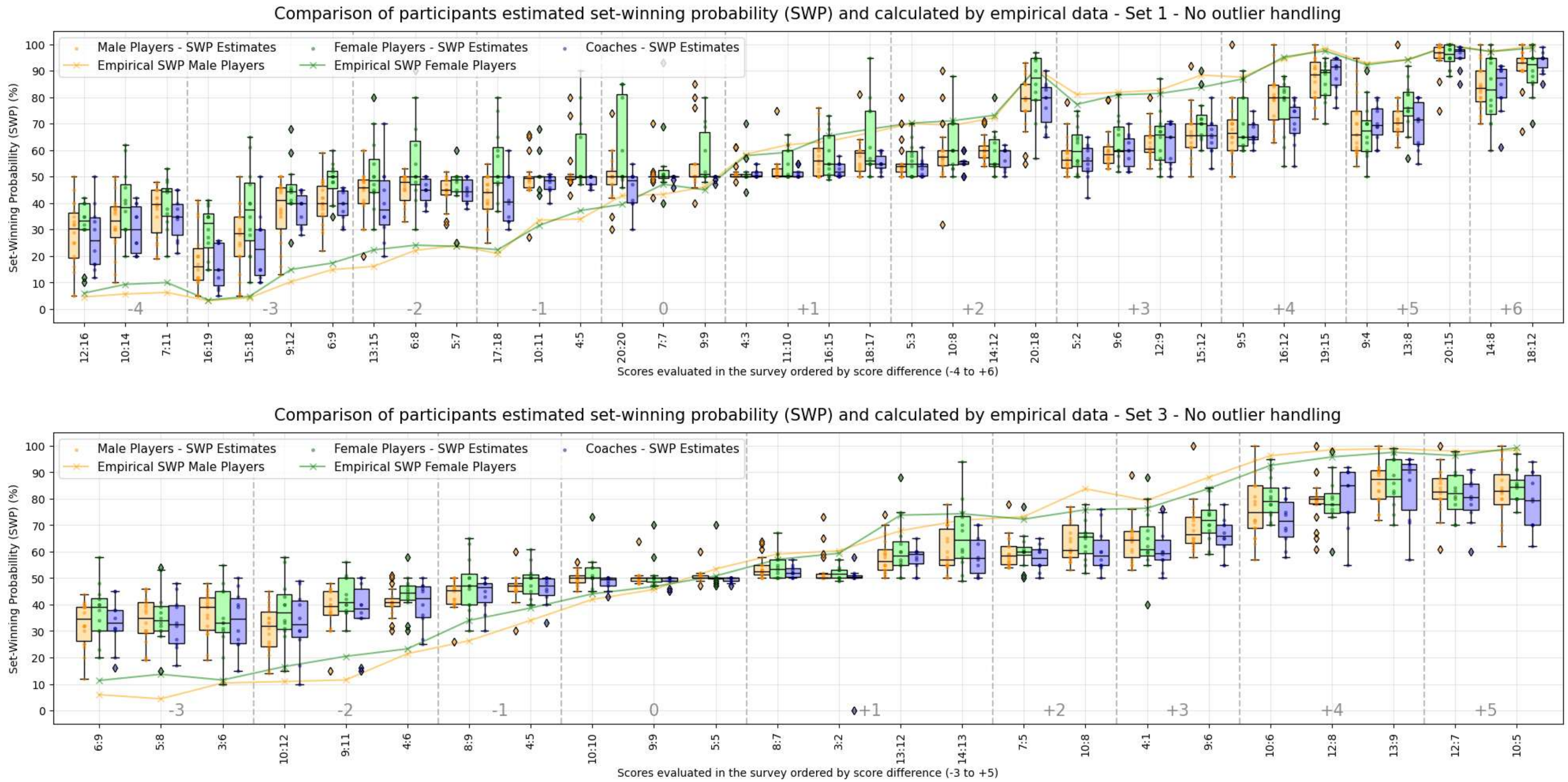


*Figure S12: Participants estimated SWPs compared to empirical calculated SWPs for each evaluated score in the first set (top) and the third set (bottom) of the survey with no outlier handling. Scores on the y-axis are ordered by the score difference of each score for better readability and do not represent the order in the survey. For coaches, both sexes are displayed together, as there is only one female coach in the sample.*

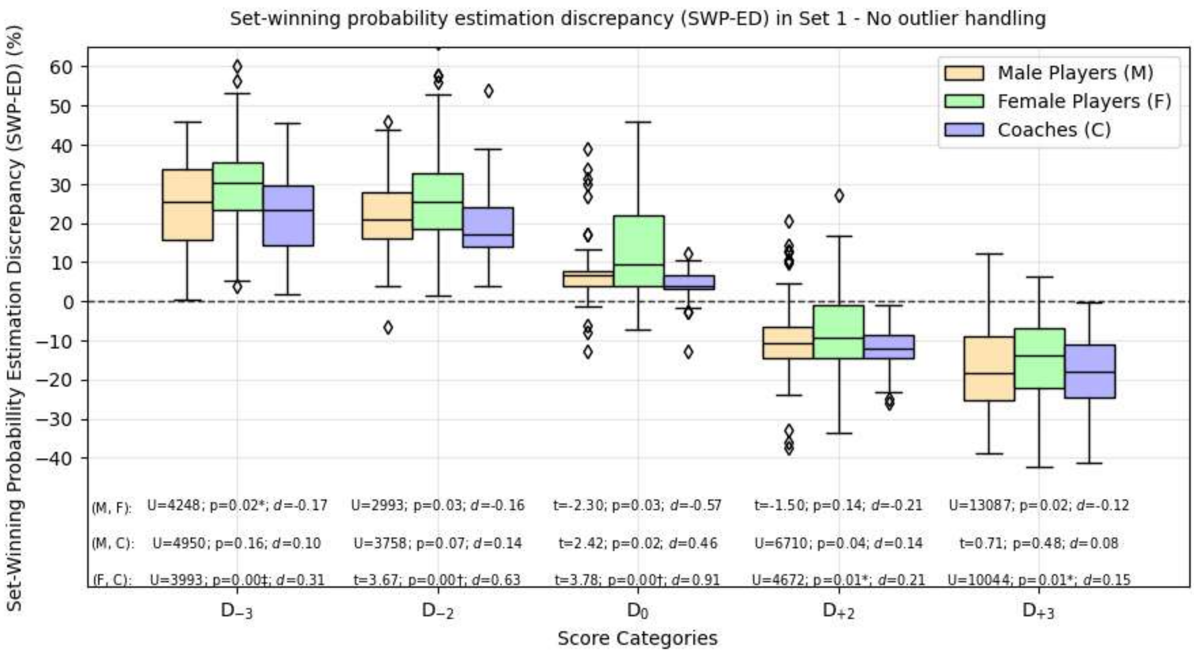


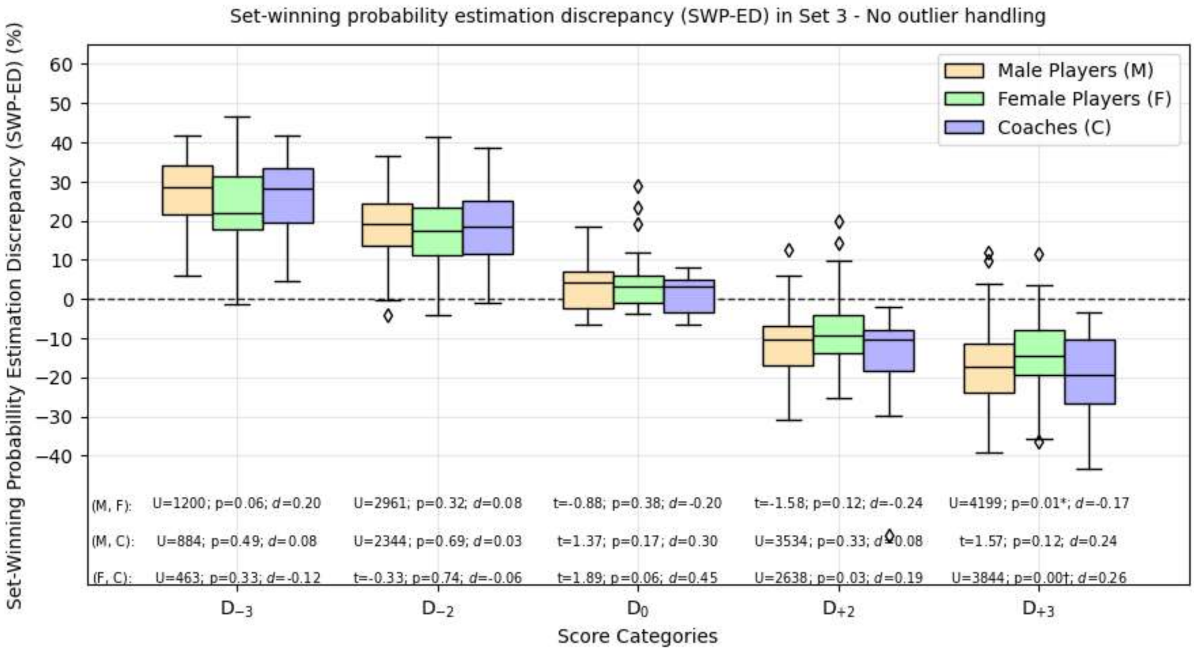


*Figure S13: Set-winning probability estimation discrepancies (SWP-ED) of participants in the first set (top) and the third set (bottom) across five score categories with no outlier handling. The red dotted line indicates perfect estimation without any discrepancy, whereas estimation above indicates over- and below underestimation of the SWP. The SWP-ED is calculated as the difference between a participant's SWP estimate and the empirically calculated SWP for the corresponding score. Independent t-tests or Man-Whitney-U tests were conducted for group comparisons within each score category. To account for multiple comparisons, a Bonferroni correction was applied, adjusting the significance level to α / m, where m is the number of tests conducted per score category. Significant results are marked with asterisk (*, p < .05 / m), dagger (†, p < .01 / m), or double dagger (‡, p < .001 / m).*

*Table S11: Pearson correlation (r) results of Linear Regression models for each participant group (M, F, C), score category, and assessed decision-making tendency as independent variable with no outlier handling.*

| | Optimism | | | Pessimism | | | Confirmation Bias | | | Sunk Cost Fallacy | | | LOT-R | | |
|---|---|---|---|---|---|---|---|---|---|---|---|---|---|---|---|
| | M | F | C | M | F | C | M | F | C | M | F | C | M | F | C |
| $D_{-3}$ | **.17*** | **.34‡** | **.30†** | **-.29‡** | **-.32‡** | -.07 | -.08 | **.47‡** | **.35‡** | -.03 | .13 | -.00 | **.26‡** | **.34‡** | **.26†** |
| $D_{-2}$ | **.20†** | .17 | **.40‡** | **-.26‡** | -.15 | -.13 | -.08 | **.31‡** | **.42‡** | .03 | -.06 | .11 | **.27‡** | .16 | **.37‡** |
| $D_0$ | .12 | .06 | .22 | **-.19*** | -.04 | .10 | .12 | .08 | .23 | .12 | -.06 | .13 | .18 | .05 | .09 |
| $D_{+2}$ | .06 | **.23†** | -.08 | -.11 | -.11 | -.09 | .05 | .15 | -.10 | **.13*** | -.01 | .04 | .09 | **.17*** | .00 |
| $D_{+3}$ | -.00 | **.25‡** | **-.22†** | .01 | -.13 | -.01 | .09 | **.20†** | -.07 | .08 | -.00 | .08 | -.01 | **.19†** | **-.15*** |

*Note: In bold, an asterisk (*, $p < .05$), dagger (†, $p < .01$), or double dagger (‡, $p < .001$) indicate significant Pearson correlations. For trailing scenarios ($D_{-3}$ and $D_{-2}$), a negative correlation indicates that higher values of the independent variable led to better estimates, as participants tended to overestimate the SWP. For leading scenarios ($D_{+2}$ and $D_{+3}$), a negative correlation suggests more estimation error or higher underestimation if the independent variable is higher, as participants in our survey predominantly underestimated the SWP.*

On the next pages, Figure S14 shows the single linear regressions with no outlier handling.

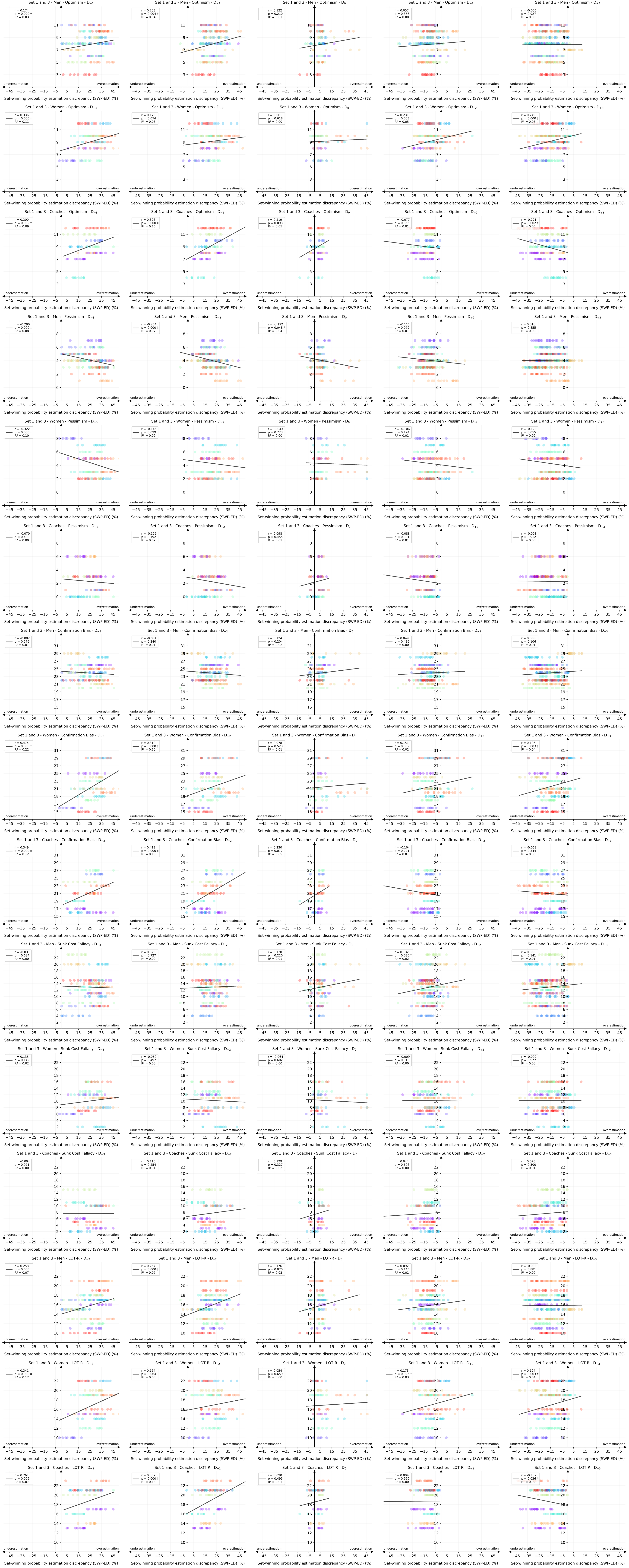

Set 1 and 3 - Men - Optimism - $D_{-3}$
r = 0.174, p = 0.020 *, R² = 0.03
Set 1 and 3 - Men - Optimism - $D_{-2}$
r = 0.203, p = 0.004 †, R² = 0.04
Set 1 and 3 - Men - Optimism - $D_0$
r = 0.122, p = 0.210, R² = 0.01
Set 1 and 3 - Men - Optimism - $D_{+2}$
r = 0.057, p = 0.366, R² = 0.00
Set 1 and 3 - Men - Optimism - $D_{+3}$
r = -0.005, p = 0.927, R² = 0.00
Set 1 and 3 - Women - Optimism - $D_{-3}$
r = 0.336, p = 0.000 ‡, R² = 0.11
Set 1 and 3 - Women - Optimism - $D_{-2}$
r = 0.170, p = 0.054, R² = 0.03
Set 1 and 3 - Women - Optimism - $D_0$
r = 0.061, p = 0.618, R² = 0.00
Set 1 and 3 - Women - Optimism - $D_{+2}$
r = 0.231, p = 0.003 †, R² = 0.05
Set 1 and 3 - Women - Optimism - $D_{+3}$
r = 0.249, p = 0.000 ‡, R² = 0.06
Set 1 and 3 - Coaches - Optimism - $D_{-3}$
r = 0.300, p = 0.002 †, R² = 0.09
Set 1 and 3 - Coaches - Optimism - $D_{-2}$
r = 0.396, p = 0.000 ‡, R² = 0.16
Set 1 and 3 - Coaches - Optimism - $D_0$
r = 0.219, p = 0.093, R² = 0.05
Set 1 and 3 - Coaches - Optimism - $D_{+2}$
r = -0.077, p = 0.365, R² = 0.01
Set 1 and 3 - Coaches - Optimism - $D_{+3}$
r = -0.221, p = 0.002 †, R² = 0.05
Set 1 and 3 - Men - Pessimism - $D_{-3}$
r = -0.290, p = 0.000 ‡, R² = 0.08
Set 1 and 3 - Men - Pessimism - $D_{-2}$
r = -0.264, p = 0.000 ‡, R² = 0.07
Set 1 and 3 - Men - Pessimism - $D_0$
r = -0.192, p = 0.048 *, R² = 0.04
Set 1 and 3 - Men - Pessimism - $D_{+2}$
r = -0.111, p = 0.079, R² = 0.01
Set 1 and 3 - Men - Pessimism - $D_{+3}$
r = 0.010, p = 0.855, R² = 0.00
Set 1 and 3 - Women - Pessimism - $D_{-3}$
r = -0.322, p = 0.000 ‡, R² = 0.10
Set 1 and 3 - Women - Pessimism - $D_{-2}$
r = -0.146, p = 0.099, R² = 0.02
Set 1 and 3 - Women - Pessimism - $D_0$
r = -0.043, p = 0.723, R² = 0.00
Set 1 and 3 - Women - Pessimism - $D_{+2}$
r = -0.106, p = 0.174, R² = 0.01
Set 1 and 3 - Women - Pessimism - $D_{+3}$
r = -0.128, p = 0.055, R² = 0.02
Set 1 and 3 - Coaches - Pessimism - $D_{-3}$
r = -0.070, p = 0.490, R² = 0.00
Set 1 and 3 - Coaches - Pessimism - $D_{-2}$
r = -0.125, p = 0.192, R² = 0.02
Set 1 and 3 - Coaches - Pessimism - $D_0$
r = 0.098, p = 0.455, R² = 0.01
Set 1 and 3 - Coaches - Pessimism - $D_{+2}$
r = -0.088, p = 0.301, R² = 0.01
Set 1 and 3 - Coaches - Pessimism - $D_{+3}$
r = -0.008, p = 0.912, R² = 0.00
Set 1 and 3 - Men - Confirmation Bias - $D_{-3}$
r = -0.082, p = 0.276, R² = 0.01
Set 1 and 3 - Men - Confirmation Bias - $D_{-2}$
r = -0.084, p = 0.240, R² = 0.01
Set 1 and 3 - Men - Confirmation Bias - $D_0$
r = 0.124, p = 0.204, R² = 0.02
Set 1 and 3 - Men - Confirmation Bias - $D_{+2}$
r = 0.049, p = 0.436, R² = 0.00
Set 1 and 3 - Men - Confirmation Bias - $D_{+3}$
r = 0.088, p = 0.106, R² = 0.01
Set 1 and 3 - Women - Confirmation Bias - $D_{-3}$
r = 0.474, p = 0.000 ‡, R² = 0.22
Set 1 and 3 - Women - Confirmation Bias - $D_{-2}$
r = 0.310, p = 0.000 ‡, R² = 0.10
Set 1 and 3 - Women - Confirmation Bias - $D_0$
r = 0.078, p = 0.523, R² = 0.01
Set 1 and 3 - Women - Confirmation Bias - $D_{+2}$
r = 0.151, p = 0.052, R² = 0.02
Set 1 and 3 - Women - Confirmation Bias - $D_{+3}$
r = 0.196, p = 0.003 †, R² = 0.04
Set 1 and 3 - Coaches - Confirmation Bias - $D_{-3}$
r = 0.349, p = 0.000 ‡, R² = 0.12
Set 1 and 3 - Coaches - Confirmation Bias - $D_{-2}$
r = 0.419, p = 0.000 ‡, R² = 0.18
Set 1 and 3 - Coaches - Confirmation Bias - $D_0$
r = 0.230, p = 0.077, R² = 0.05
Set 1 and 3 - Coaches - Confirmation Bias - $D_{+2}$
r = -0.104, p = 0.221, R² = 0.01
Set 1 and 3 - Coaches - Confirmation Bias - $D_{+3}$
r = -0.069, p = 0.344, R² = 0.00
Set 1 and 3 - Men - Sunk Cost Fallacy - $D_{-3}$
r = -0.031, p = 0.684, R² = 0.00
Set 1 and 3 - Men - Sunk Cost Fallacy - $D_{-2}$
r = 0.025, p = 0.727, R² = 0.00
Set 1 and 3 - Men - Sunk Cost Fallacy - $D_0$
r = 0.120, p = 0.220, R² = 0.01
Set 1 and 3 - Men - Sunk Cost Fallacy - $D_{+2}$
r = 0.132, p = 0.036 *, R² = 0.02
Set 1 and 3 - Men - Sunk Cost Fallacy - $D_{+3}$
r = 0.080, p = 0.141, R² = 0.01
Set 1 and 3 - Women - Sunk Cost Fallacy - $D_{-3}$
r = 0.142, R² = 0.02
Set 1 and 3 - Women - Sunk Cost Fallacy - $D_{-2}$
r = -0.060, p = 0.497, R² = 0.00
Set 1 and 3 - Women - Sunk Cost Fallacy - $D_0$
r = -0.064, p = 0.602, R² = 0.00
Set 1 and 3 - Women - Sunk Cost Fallacy - $D_{+2}$
r = -0.009, p = 0.910, R² = 0.00
Set 1 and 3 - Women - Sunk Cost Fallacy - $D_{+3}$
r = 0.002, p = 0.977, R² = 0.00
Set 1 and 3 - Coaches - Sunk Cost Fallacy - $D_{-3}$
r = -0.004, p = 0.971, R² = 0.00
Set 1 and 3 - Coaches - Sunk Cost Fallacy - $D_{-2}$
r = 0.110, p = 0.254, R² = 0.01
Set 1 and 3 - Coaches - Sunk Cost Fallacy - $D_0$
r = 0.129, p = 0.327, R² = 0.02
Set 1 and 3 - Coaches - Sunk Cost Fallacy - $D_{+2}$
r = 0.044, p = 0.606, R² = 0.00
Set 1 and 3 - Coaches - Sunk Cost Fallacy - $D_{+3}$
r = 0.076, p = 0.300, R² = 0.01
Set 1 and 3 - Men - LOT-R - $D_{-3}$
r = 0.258, p = 0.000 ‡, R² = 0.07
Set 1 and 3 - Men - LOT-R - $D_{-2}$
r = 0.267, p = 0.000 ‡, R² = 0.07
Set 1 and 3 - Men - LOT-R - $D_0$
r = 0.176, p = 0.070, R² = 0.03
Set 1 and 3 - Men - LOT-R - $D_{+2}$
r = 0.092, p = 0.145, R² = 0.01
Set 1 and 3 - Men - LOT-R - $D_{+3}$
r = -0.008, p = 0.881, R² = 0.00
Set 1 and 3 - Women - LOT-R - $D_{-3}$
r = 0.341, p = 0.000 ‡, R² = 0.12
Set 1 and 3 - Women - LOT-R - $D_{-2}$
r = 0.164, p = 0.064, R² = 0.03
Set 1 and 3 - Women - LOT-R - $D_0$
r = 0.054, p = 0.659, R² = 0.00
Set 1 and 3 - Women - LOT-R - $D_{+2}$
r = 0.173, p = 0.025 *, R² = 0.03
Set 1 and 3 - Women - LOT-R - $D_{+3}$
r = 0.194, p = 0.003 †, R² = 0.04
Set 1 and 3 - Coaches - LOT-R - $D_{-3}$
r = 0.261, p = 0.009 †, R² = 0.07
Set 1 and 3 - Coaches - LOT-R - $D_{-2}$
r = 0.367, p = 0.000 ‡, R² = 0.13
Set 1 and 3 - Coaches - LOT-R - $D_0$
r = 0.090, p = 0.495, R² = 0.01
Set 1 and 3 - Coaches - LOT-R - $D_{+2}$
r = 0.004, p = 0.960, R² = 0.00
Set 1 and 3 - Coaches - LOT-R - $D_{+3}$
r = -0.152, p = 0.036 *, R² = 0.02
underestimation
overestimation
Set-winning probability estimation discrepancy (SWP-ED) (%)

# 6. Comparison of linear regression results with different outlier handling approaches

To assess the robustness of our findings, we compared the results obtained using three different outlier correction methods: (1) our customized approach, (2) standardized winsorizing, and (3) no outlier exclusion. The comparisons show no substantial discrepancies between the selected customized method and the alternative approaches. For the simple linear regression models, Pearson correlations and $R^2$ values are only marginally lower when using the customized outlier handling technique. In summary, the chosen customized outlier handling method has no meaningful impact on the interpretation of the results. If anything, the approach tends to produce more conservative significance levels, thereby strengthening confidence in the robustness of the observed effects. On the following page in Table S12, a comparison of all linear regression results with different outlier handling approaches is shown.

*Table S12: Comparison of single linear regression analyses results with different outlier approaches assessed*

| | | Optimism | | | Pessimism | | | Confirmation Bias | | | Sunk Cost Fallacy | | | LOT-R | | |
|---|---|---|---|---|---|---|---|---|---|---|---|---|---|---|---|---|
| | | M | F | C | M | F | C | M | F | C | M | F | C | M | F | C |
| customized outlier handling | $D_{-3}$ | .17 * | .32 *** | .30 ** | -.28 *** | -.31 *** | -.07 | -.07 | .46 *** | .35 *** | -.03 | .15 | -.00 | .25 *** | .33 *** | .26 ** |
| | $D_{-2}$ | .20 ** | .12 | .43 *** | -.24 *** | -.10 | -.10 | -.09 | .27 ** | .39 *** | .01 | -.03 | .10 | .25 *** | .11 | .37 *** |
| | $D_{0}$ | .16 | .08 | .22 | -.17 | -.05 | .10 | .01 | .10 | .23 | .02 | -.05 | .13 | .19 | .07 | .09 |
| | $D_{+2}$ | .06 | .23 ** | -.09 | -.14 * | -.09 | -.16 | .05 | .18 * | -.04 | .14 * | -.01 | .09 | .11 | .16 * | .04 |
| | $D_{+3}$ | .00 | .25 *** | -.21 ** | -.01 | -.13 | -.00 | .10 | .19 ** | -.10 | .09 | -.01 | .07 | .01 | .19 ** | -.15 * |
| Winsorizing approach | $D_{-3}$ | .17 * | .33 *** | .30 ** | -.29 *** | -.32 *** | -.07 | -.08 | .47 *** | .35 *** | -.03 | .13 | -.00 | .26 *** | .34 *** | .26 ** |
| | $D_{-2}$ | .20 ** | .17 | .40 *** | -.27 *** | -.14 | -.13 | -.07 | .31 *** | .42 *** | .04 | -.06 | .11 | .27 *** | .16 | .37 *** |
| | $D_{0}$ | .12 | .06 | .22 | -.17 | -.04 | .09 | .11 | .09 | .22 | .10 | -.07 | .12 | .16 | .05 | .09 |
| | $D_{+2}$ | .05 | .23 ** | -.08 | -.08 | -.10 | -.11 | .06 | .15 | -.09 | .13 * | -.02 | .06 | .07 | .17 * | .01 |
| | $D_{+3}$ | -.00 | .25 *** | -.22 ** | .01 | -.13 | -.01 | .06 | .20 ** | -.07 | .06 | -.00 | .07 | -.01 | .19 ** | -.15 * |
| No outlier handling | $D_{-3}$ | .17 * | .34 *** | .30 ** | -.29 *** | -.32 *** | -.07 | -.08 | .47 *** | .35 *** | -.03 | .13 | -.00 | .26 *** | .34 *** | .26 ** |
| | $D_{-2}$ | .20 ** | .17 | .40 *** | -.26 *** | -.15 | -.13 | -.08 | .31 *** | .42 *** | .03 | -.06 | .11 | .27 *** | .16 | .37 *** |
| | $D_{0}$ | .12 | .06 | .22 | -.19 * | -.04 | .10 | .12 | .08 | .23 | .12 | -.06 | .13 | .18 | .05 | .09 |
| | $D_{+2}$ | .06 | .23 ** | -.08 | -.11 | -.11 | -.09 | .05 | .15 | -.10 | .13 * | -.01 | .04 | .09 | .17 * | .00 |
| | $D_{+3}$ | -.00 | .25 *** | -.22 ** | .01 | -.13 | -.01 | .09 | .20 ** | -.07 | .08 | -.00 | .08 | -.01 | .19 ** | -.15 * |

*Note: Asterisks (*, $p < .05$), (**, $p < .01$), or (***, $p < .001$) indicate significant Pearson correlations. For readability, yellow marked areas differ to the selected customized outlier handling approach in the manuscript*
